# Comparing Gas and Inhaled Pharmacologic Interventions During Ex Vivo Lung Perfusion: A Systematic Review

**DOI:** 10.1097/TXD.0000000000002012

**Published:** 2026-09-16

**Authors:** Maira Machhiwala, Ryaan EL-Andari, Parham Hassanzadeh, Adrien Lam, Daniel Gutierrez, Darren H. Freed, Jayan Nagendran

**Affiliations:** 1 Faculty of Medicine and Dentistry, University of Alberta, Edmonton, AB, Canada.; 2 Division of Cardiac Surgery, Department of Surgery, University of Alberta, Edmonton, AB, Canada.; 3 Department of Surgery, University of Alberta, Edmonton, AB, Canada.

## Abstract

**Background.:**

Ex vivo lung perfusion (EVLP) has expanded the use of donor lungs by enabling ex vivo assessment, preservation, and rehabilitation before transplantation. Despite these advances, ischemia-reperfusion injury, inflammatory activation, pulmonary edema, and impaired lung mechanics continue to compromise graft quality and contribute to primary graft dysfunction. Inhaled and gaseous interventions have emerged as potential adjunctive therapies to improve donor lung preservation during EVLP; however, a standardized protocol has not yet been established.

**Methods.:**

PubMed and Embase were used to search human and animal studies evaluating inhaled and gaseous interventions administered during and after EVLP. Articles published from the database’s inception to September 22, 2025, were analyzed.

**Results.:**

Among the interventions examined, 10% carbon dioxide, carbon monoxide (CO), 40% oxygen (O_2_), hydrogen, 40 ppm nitric oxide (NO) with 60% O_2_, 2% sevoflurane with 40%–50% O_2_, 2-S-amino-6-boronohexanoic acid, fasudil, and β_2_-adrenoreceptor agonists most effectively improved lung function. Interestingly, improvements from hydrogen and CO were observed after transplantation, rather than during EVLP, suggesting more persistent or delayed protective effects. Further optimized parameters were observed when moderate O_2_ concentrations (40%–60%) were combined with either CO, NO, or sevoflurane, compared with O_2_ alone. In contrast, argon, xenon, N-acetylcysteine, sevoflurane or NO with 6% O_2_, and liquid ventilation demonstrated minimal benefit.

**Conclusions.:**

Inhaled interventions can attenuate both mechanical damage and inflammatory pathways, particularly when used in tailored combinations with 40%–60% O_2_. Future research should prioritize standardizing protocols, optimizing gas composition and timing, and assessing long-term posttransplant outcomes to translate these findings into clinical practice.

## INTRODUCTION

For patients with end-stage lung disease refractory to medical management, lung transplantation remains the gold standard treatment. However, the availability of suitable donor lungs remains limited and must meet strict acceptance criteria, including adequate oxygenation, acceptable bronchoscopic findings, and limited ischemic time before transplantation.^1^ Additionally, even when a donor lung has been transplanted into a recipient, primary graft dysfunction (PGD) contributes to early mortality in 30%–80% of lung transplant recipients in the first 48–72 h.^2,3^

Several medical gases and inhaled therapies, such as hydrogen (H_2_) and carbon monoxide (CO), are being evaluated for their known chemical properties in reducing inflammatory markers, limiting oxidative damage, and decreasing pulmonary edema formation.^4-6^ These properties have generated interest in their application during ex vivo lung perfusion (EVLP), a platform that preserves, evaluates, and rehabilitates donor lungs through normothermic ventilation and perfusion before transplantation. Although EVLP has expanded the donor pool by improving donor lung assessment and enabling therapeutic intervention, challenges such as ischemia-reperfusion injury, inflammatory activation, pulmonary edema, and subsequent PGD continue to limit graft quality and posttransplant outcomes.^7-9^

A growing number of studies have evaluated inhaled and gaseous interventions during EVLP to improve donor lung preservation. However, a comprehensive comparison of these approaches has not been conducted. This systematic review examines current evidence on inhaled interventions in the ex vivo setting, focusing on their effects on inflammation, lung mechanics, and posttransplant graft function.

## MATERIALS AND METHODS

### Data Sources

This review followed the Preferred Reporting Items for Systematic Reviews and Meta-Analyses guidelines.^10^ PubMed and Embase searches were completed, searching for English-language studies from their inception to September 22, 2025. Search terms included: ((gas) OR (hydrogen) OR (bronchodilator) OR (inhalation) OR (nitric oxide) OR (nitrogen) OR (carbon monoxide) OR (ventilation)) AND ((Ex vivo lung perfusion) OR (Ex situ lung perfusion) OR (EVLP) OR (ESLP) OR (extracorporeal lung perfusion)). The review was registered on Open Science Framework under 10.17605/OSF.IO/8E4CR.

### Study Selection

**FIGURE 1.**
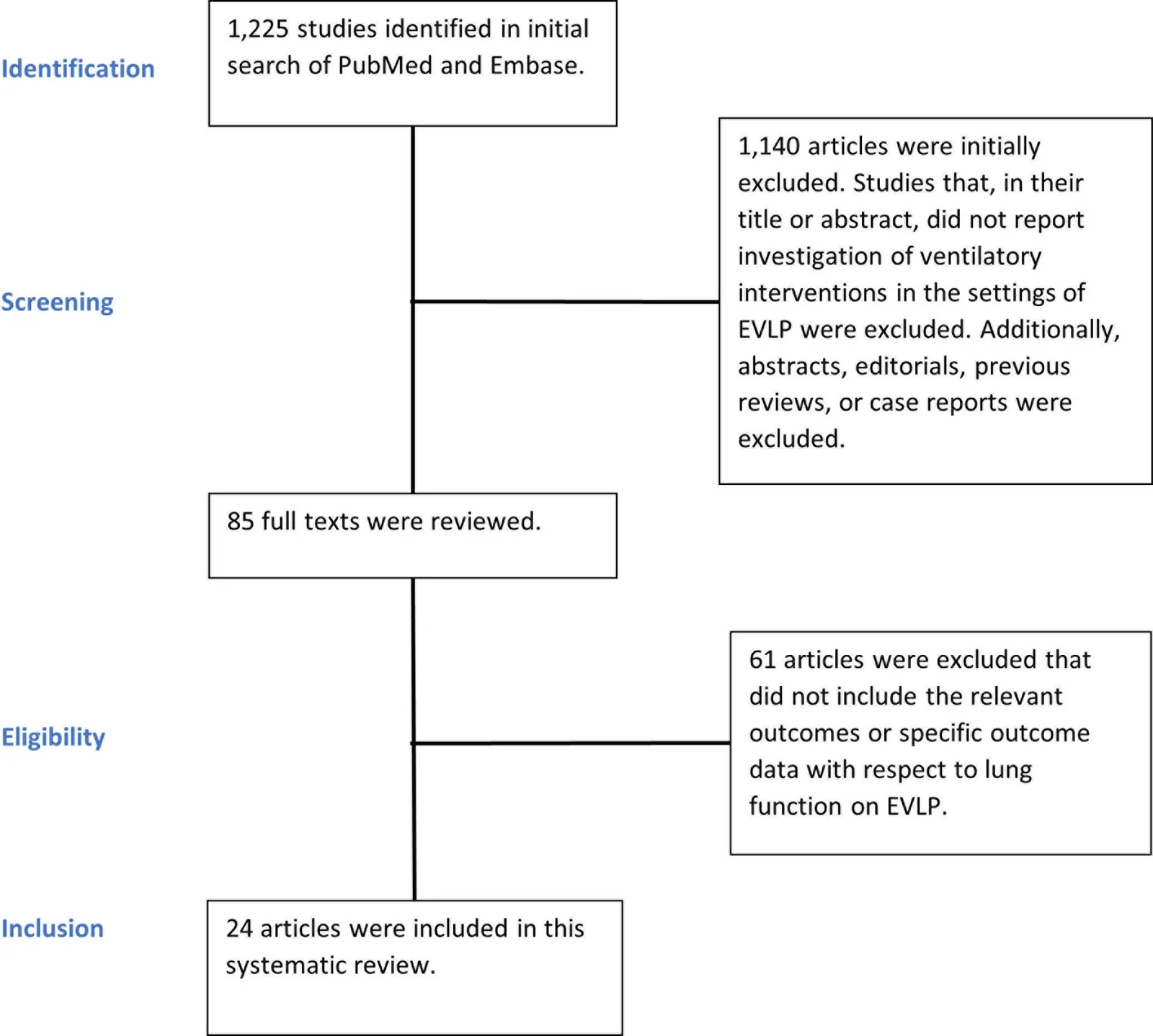
Preferred Reporting Items for Systematic Reviews and Meta-Analyses (PRISMA) flow diagram for study inclusion. EVLP, ex vivo lung perfusion.

The included studies focused on EVLP using human or animal models to study the effects of various ventilatory modifications or administration of inhaled agents during EVLP. The review process excluded the following study types: reviews, case reports, editorials, abstracts without full text, and studies unrelated to inhaled medications or gas administration during EVLP. Two authors screened and reviewed all articles. Any disagreement was settled through a third author. Figure 1 shows the summary of the literature selection process.

### Outcomes

The main outcomes of interest examined included: weight gain, compliance, pulmonary artery pressure (PAP), pulmonary vascular resistance (PVR), airway pressure, oxygenation, inflammatory markers, edema formation, and/or markers of cell death.

### Risk of Bias

**FIGURE 2.**
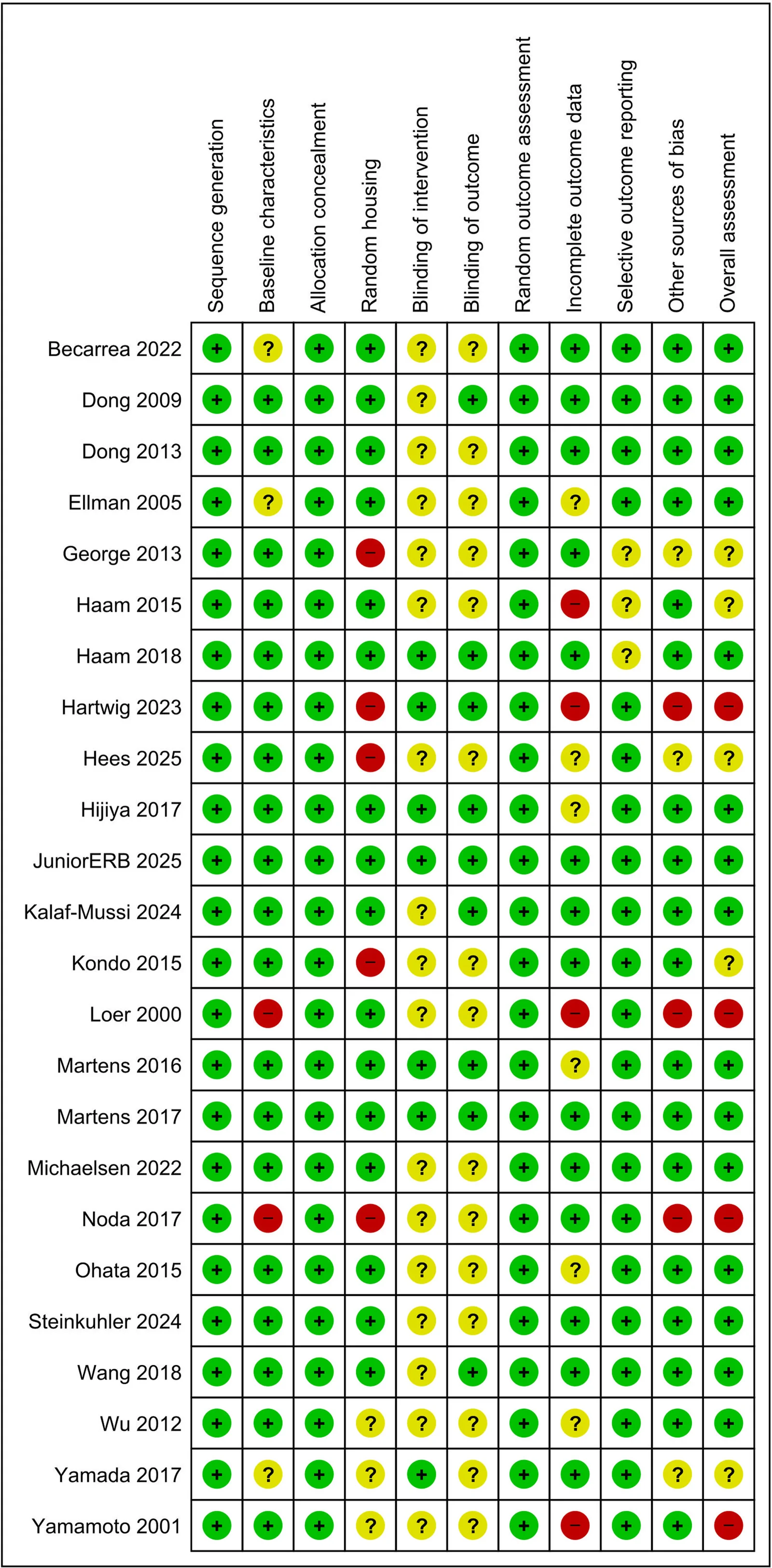
Risk of bias assessment based on SYRCLE’s (SYstematic Review Centre for Laboratory animal Experimentation) risk of bias tool for animal studies.

The risk of bias was evaluated using SYRCLE’s (SYstematic Review Centre for Laboratory animal Experimentation) tool for animal studies.^11^ This tool examines potential sources of bias, including selection, detection, performance, and reporting bias, as well as confounding factors. A summary of the assessment is presented in Figure 2.

## RESULTS

### Overview of Included Studies

Our literature search yielded 1225 articles and 85 full texts, resulting in 25 included studies in our review. The duration of EVLP ranged from 1 to 12 h, and posttransplantation follow-up ranged from 1 to 4 h. Of the included studies, 10 used rodents, 8 used pigs, 3 used rabbits, 2 used dogs, 1 used sheep, and 2 used human lungs. Of the total sample size of 321, 39 received carbon dioxide (CO_2_), 12 received argon (Ar), 5 received xenon (Xe), 22 received nitric oxide (NO), 7 received 2-S-amino-6-boronohexanoic acid (ABH), 12 received β_2_-adrenergic agonist, 11 received CO, 15 received H_2_ gas, 14 received sevoflurane, 13 received liquid ventilation, 6 received fasudil, 6 received N-acetylcysteine (NAC), 10 received 21%–24% oxygen gas (O_2_), 10 received 100% O_2_, and 128 were used as controls. These numbers may not represent the exact total, as Noda et al^11^ did not specify their sample sizes. Additionally, although Noda et al^11^ delivered various concentrations of O_2_ through a deoxygenator rather than via direct inhalation, the study was included because it evaluated the effects of different O_2_ concentrations during EVLP. This was particularly relevant, as O_2_ was used in combination with several inhaled and gaseous interventions included in our review, enabling assessment of whether observed benefits were attributable to O_2_ concentration itself or to synergistic effects of combined therapies. Table 1 entails details about our included studies. Table 2 summarizes the primary outcomes, including edema formation, lung function, airway pressure, lactate levels, and histological lung injury score. Finally, markers of inflammation, apoptosis, and oxidative stress are reported in Table 3.

**TABLE 1.** Summary of included studies

Study name	Model/population	Groups	Duration of perfusion and posttransplantation observation period	Inhaled medication or gas used and gas mixture	Mode of administration	Rate of administration
Becerra et al,^12^ 2022	Adult male Sprague-Dawley rats (250–450 g) underwent single left lung EVLP	Liquid ventilation: 6 Control: 6 Total: 12	6 h	Liquid (not specified) or gas (control) ventilation during EVLP	Ventilation	Administered during EVLP, tidal volume of 6 mL/kg
Dong et al,^13^ 2009	Male Sprague-Dawley rats (350–450 g) underwent heart-lung block procurement, double lung EVLP, and left lung transplantation	NO: 6 Control: 6 Total: 12	1 h EVLP + 1 h posttransplantation	60% O_2_ alone or 60% O_2_ with 40 ppm N)	Ventilation	Administered for 1 h postcardiac arrest and during EVLP, tidal volume of 0.75 mL/100 g, rate 60/min
Dong et al,^14^ 2013	Male Sprague-Dawley rats (350–450 g) underwent heart-lung block procurement, double lung EVLP, and left lung transplantation	CO: 6 Control: 6 Total: 12	1 h EVLP + 1 h posttransplantation	60% O_2_ alone or 60% O_2_ with 500 ppm CO	Ventilation	Administered for 1 h postcardiac arrest and during EVLP, tidal volume of 0.75 mL/100 g and rate of 70/min
Ellman et al,^15^ 2005	Both sexes of New Zealand white rabbits (3–4.5 kg) underwent en bloc procurement	21%–24% O_2_: 10 100% O_2_: 10 Total: 20	3 h	Either 100% FiO_2_ or room air	Ventilation	Administered during EVLP, 8–12 mL/kg with 2.5 cm H_2_O of positive end-expiratory pressure at a rate of 20 breaths/min.
George et al,^16^ 2013	7 human double lungs split into 14 single lungs individually undergoing EVLP	Delayed EVLP (4 h CS) + early ABH: 3 Delayed EVLP + late ABH: 4 Total: 7	4 h	ABH dissolved in normal saline to a concentration of 25 uM and nebulized Lungs were ventilated with room air with an FiO_2_ of 21%, and transiently 100% FiO_2_ every hour Sweep gas flow into oxygenator for deoxygenation was 6% O_2_, 8% CO_2_, and 86% N_2_ (all groups)	Nebulized	Early ABH: Over 10 min of ventilation at the start of EVLP Late ABH: After 3 h of EVLP
Haam et al,^17^ 2015	40 kg Yorkshire female pigs undergoing EVLP	Control: 5 Hydrogen gas: 5 Total: 10	4 h	Room air (21% FiO_2_) 2% H_2_ mixed with room air (21% FiO_2_) Sweep gas flow into oxygenator for deoxygenation was 6% O_2_, 8% CO_2_, and 86% N_2_ (all groups)	Ventilation	Administered during EVLP, Continuous tidal volume of 7 mL/kg, a respiration rate of 7/min
Haam et al,^18^ 2018	40 kg Yorkshire female pigs undergoing EVLP, then orthotopic left lung graft transplant	Control: 5 Hydrogen gas: 5 Total: 10	4 h EVLP + 3 h posttransplantation	Before: FiO_2_ of 50% During: Room air (21% FiO_2_) 2% H_2_ mixed with room air (21% FiO_2_) Sweep gas flow into oxygenator for deoxygenation was 6% O_2_, 8% CO_2_, and 86% N_2_ (all groups) After: FiO_2_ of 50%	Ventilation	Before EVLP: 10 mL/kg tidal volume, respiration rate 16–18/min During EVLP: 7 mL/kg tidal volume, respiration rate 7/min After EVLP: 10 mL/kg tidal volume, respiration rate 12/min
Hartwig et al,^19^ 2023	Bilateral donor lungs declined for transplantation; blinded study with open-label study	Control (perfusate alone): 8 gNO (via perfusate): 8 iNO (substudy; via ventilator): 4 Total: 20	6 h	6% O_2_, 8% CO_2_, 86% N_2_ (into oxygenator) 80 ppm gNO + above (into oxygenator) 20 ppm into ventilation system in addition to 6% O_2_, 8% CO_2_, 86% N_2_ into oxygenator	Ventilation	Not specified
Hees et al,^20^ 2025	Bilateral lungs of male Sprague-Dawley rats, isolated, ventilated, and perfused	Control: 6 Argon: 7 Total: 13	70 min	65% N_2_, 5% CO_2_, 30% O_2_ 65% Ar, 5% CO_2_, 30% O_2_	Ventilation	Administered during EVLP, 10 mL/kg tidal volume, respiration rate 30/min
Hijiya et al,^21^ 2017	Beagle dogs (10–12.5 kg) underwent double-lung EVLP, then left lung transplant	β_2_-adrenergic agonist: 5 Control: 5 Total: 10	2 h EVLP + 4 h posttransplantation	1400 mg of the β_2_-adrenergic receptor agonist, procaterol hydrochloride hydrate (0.01% procaterol inhalation solution [14 mL])	Nebulized	Every 30 min (4 times during EVLP)
Junior et al,^22^ 2025	Male, domestic Yorkshire pigs (28.4–41.1 kg) underwent double lung EVLP	Intrapleural topical cooling + sevoflurane: 4 Intrapleural topical cooling: 4 Control: 4 Total: 12	6 h	Intrapleural temperature (10–15 °C), combined with 2% sevoflurane and FiO_2_ = 50%	Inhaled via AnaConDa device (10 mL/h)	2% sevoflurane during ventilation via an AnaConDa device (10 mL/h) for 30 min before EVLP
Kalaf-Mussi et al,^23^ 2024	Yorkshire male domestic pigs (28–35 kg) underwent double lung EVLP and left lung transplant	CO: 5 Control: 5 Total: 10	6 h EVLP + 4 h posttransplantation	Custom designed gas cylinder (CO 500 ppm, 8% CO_2_, 6% O_2_, and balanced N_2_) and regulator (100 PSI max, Prostar Platinum, Praxair, Brampton, ON, Canada)	Ventilation	During the first hour of EVLP
Kondo et al,^24^ 2015	Beagles (9–11 kg) underwent double lung EVLP	β_2_-adrenoreceptor agonist: 7 Control: 6 Total:13	4 h	β_2_-adrenoreceptor agonist (0.01% procaterol [3.5 mL] inhalation solution [350 mg procaterol])	Inhaled	Provided between 20 and 50 min into starting EVLP (30-min duration)
Loer et al,^25^ 2000	Both sexes of New Zealand White rabbits (2.6–3.1 kg) underwent en bloc removal and double lung EVLP	Perfluorocarbon: 7 Control: 7 Total: 14	Not specified	Perfluorocarbon: chemical structure C_8_F_18_	Ventilation	Administered during EVLP, continuous 15 mL/kg body weight
Martens et al,^26^ 2016	Topig 20 male domestic pigs (36–42 kg) underwent double lung EVLP Positive control, Argon, Xenon groups underwent 120 min of warm ischemia Negative control: no warm ischemia	Argon: 5 Xenon: 5 Positive control: 5 Negative control: 5 Total: 20	6 h	Ar or Xenon or 70% N_2_ (positive control) 30% O_2_ in all groups	Ventilation	Administered during EVLP, tidal volume 7 mL/kg, respiration rate 7/min
Martens et al,^27^ 2017	Topig 20 male domestic pigs (mean weight 35.7 kg) underwent double-lung EVLP	Argon: 6 Control: 6 Total: 12	4 h	21% O_2_ and 79% Ar or 79% N_2_	Ventilation	Ar: 7 animals were preventilated for 6 h with 21% O_2_ and 79% Ar Control: animals were preventilated for 6 h with 21% O_2_ and 79% N_2_ Rate in both groups: tidal volume 8 mL/kg, respiratory rate adjusted to end-tidal CO_2_, on 35–45 mm Hg
Michaelsen et al,^28^ 2022	Male Yorkshire pigs (28–35 kg) lung infected with *Pseudomonas aeruginosa*, *Staphylococcus aureus*, *Escherichia coli*, and *Burkholderia cepacia* underwent double lung EVLP	NO:4 Control: 4 Total: 8	12 h	86% N_2_, 8% CO_2_, 6% O_2_, with or without 200 ppm NO	Ventilation	Administered during EVLP, 0.8 L/min through a membrane oxygenator
Noda et al,^29^ 2014	Male Lewis rats (250–300 g) underwent heart-en-bloc EVLP for 4 h or 2 h of heart-en-bloc EVLP, followed by cold storage and lung transplantation	Hydrogen: 5 Control: 5 Total: 10	4 h	2% H_2_ mixed with room air (21% FiO_2_) Room air (21% FiO_2_)	Ventilation	Administered during EVLP; rate not specified
Noda et al,^11^ 2017	250–300 g male rats underwent Heart-en-bloc EVLP	6% O_2_ 40% O_2_ 60% O_2_ 100% O_2_ n = 6–8 per group	4 h	6%, 40%, 60%, or 100% O_2_ and 8% CO_2_ and balance N_2_	Through an oxygenator and tracheotomy	75 ml/min/250 g donor body weight.
Ohata et al,^30^ 2017	290–330 g male rats underwent heart-en-bloc EVLP	Sham: continuous perfusion (no ischemia): 6 60-min warm ischemia: 6 Fasudil (+60 min warm ischemia): 6 Total: 18	3 h	Nebulized fasudil (100 mmol/L) during first 5 min of ischemia	Nebulized	Drug in the first 5 min of warm ischemia before EVLP
Steinkühler et al,^31^ 2024	41–46 kg sheep underwent double lung EVLP	Control: 5 S-EVLP: 5 Total: 10	4 h	Sevoflurane 2% end-tidal concentration administered continuously, FiO_2_: 40%	SedaConDa ACD-S device	Start of EVLP bolus 0.5 mL, 2.8 mL/hr via SedaConDa ACD-S device during EVLP
Wang et al,^32^ 2018	Male rats 9–11 wk old, 300–350 g, underwent Heart-en-bloc EVLP	Control: 6 Sevoflurane: 2% sevoflurane during the first 30 min of EVLP: 6 Total: 12	3 h	Gas mixture for deoxygenation: 6% O_2_ + 10% CO_2_ + 84% nitrogen	Vapor 2000 Dräger inhalation	Sevoflurane was administered during the first 30 min of EVLP and stopped when mechanical ventilation was initiated
Wu et al,^33^ 2012	12-wk-old adult male Sprague-Dawley rats (350 ± 20 g) underwent double lung EVLP	Control: FiCO_2_ 5%: 6 IR FiCO_2_ 5%: 6 IR + hypercapnic acidosis FiCO_2_ 10%: 6 Total: 18	60 min	Control/IR: 5% CO_2_, 21% O_2_, and 74% N_2_ HCA: 10% CO_2_, 21% O_2_, and 69% N_2_	Tracheal cannula	Administered during EVLP: 60 cycle/min, a tidal volume of 3 mL, and an end-expiratory pressure of 1 cm H_2_O
Yamada et al,^34^ 2017	Female pigs (30–53 kg) underwent double lung EVLP followed by left lung transplantation	Control (saline inhalation): 5 NAC nebulization: 6 Total: 11	2 h EVLP followed by 1 h reperfusion after transplant, followed by 5 h isolated left-lung observation posttransplant	Fluimucil 20%, 50 mg/kg	Nebulized	50 mg/kg single dose at the beginning of EVLP
Yamamoto et al,^35^ 2001	Male Japanese albino rabbits (3.0–3.5 kg) underwent Heart-en-bloc EVLP	Alveolar CO_2_ exposure to blood-perfused lungs (FiCO_2_ 0–0.12): 5 Intravascular CO_2_ exposure to blood-perfused lungs (FiCO_2_ 0–0.12): 5 Alveolar CO_2_ exposure to buffer-perfused lungs: 6 Intravascular CO_2_ exposure to buffer-perfused lungs: 6 Hypoxia + hypercapnia (buffer-perfused lungs): 5 Total: 27	25 min	FiCO_2_ 0–0.12, 20% O_2_, 6% CO_2_, balance N_2_ (standard gas)	Alveolar exposure: treatment gas was inhaled Intravascular exposure: gas was supplied to the perfusion circuit	Respective gas condition exposed for 15 min; remaining 10 min of perfusion used standard gas at 0.9 L/min ventilation (30 mL × 30 cycles/min)

Dashes indicates that data were not available for the indicated variable. ABH, 2-S-amino-6-boronohexanoic acid; Ar, argon; CO, carbon monoxide; CS, cold storage; EVLP, ex vivo lung perfusion; FiCO_2_, fraction of inspired carbon dioxide; FiO_2_, fraction of inspired oxygen; gNO, gaseous nitric oxide; HCA, hypercapnic acidosis; iNO, inhaled nitric oxide gas; IR, ischemia-reperfusion-induced lung injury; N_2_, nitrogen; NAC, N-acetylcysteine; NO, nitric oxide; S-EVLP, sevoflurane ex vivo lung perfusion.

**TABLE 2.** Primary outcomes

Study name	Group	Edema formation, *P*	Pulmonary vascular resistance, *P*	Pulmonary artery pressure, *P*	Oxygenation (P/F ratio or oxygen, partial pressure), *P*	Dynamic compliance, *P*	Airway obliteration, *P*	Lactate, *P*	Histological lung injury score
Becerra et al,^12^ 2022	Liquid ventilation Control	W/D: NS between groups, 0.16	NS between groups, 0.11	–	P/F ratio: NS between groups, >0.05	NS between groups, 0.17	–	NS between groups, >0.05	–
Dong et al,^13^ 2009	NO Control	W/D: After EVLP: NO: reduced vs control, **<0.003** After transplantation: NS between groups, >0.05	–	–	After transplantation: P/F ratio: NO: higher vs control, **<0.001**	–	Mean Airway pressure (mm Hg): NO: lower vs control, 0.07	–	–
Dong et al,^14^ 2013	CO Control	W/D: EVLP: CO: less vs control, **0.0356** After transplantation: NS between groups, >0.05	–	–	After transplantation: P/F ratio: CO: higher vs control, **0.0058**	–	Graft airway pressure: NS between groups, 0.14	–	
Ellman et al,^15^ 2005	21%–24% O_2_ 100% O_2_	W/D: 21%–24% O_2_: lower vs 100% O_2_, **0.002**	–	NS between groups, >0.05	P/F ratio: NS between groups, >0.05	–	Peak inspiratory pressure: NS between groups, >0.05	–	–
George et al,^16^ 2013	Early ABH Late ABH	–	–	NS between groups, 0.5	PO_2_: NS between groups, >0.05	Early ABH: 34 ± 11 at baseline to 73 ± 17 ml/cm H_2_O Late ABH: from 39 ± 19 to 46 ± 23 mL/cm H_2_O, **0.001**	–	–	–
Haam et al,^17^ 2015	Control Hydrogen	W/D After EVLP: 5.715 ± 0.557 5.077 ± 0.268, **0.0400**	PVR lower in hydrogen group, **0.0111**	Lower in the hydrogen group, **0.0189**	Oxygen transfer capacity lower in the hydrogen group, >0.05	–	–	–	Lung injury severity score: 0.9 ± 0.1 0.2 ± 0.2, **0.0358**
Haam et al,^18^ 2018	Control Hydrogen	W/D After transplant: 2.833 ± 0.354 1.766 ± 0.078, >0.05	During: NS between groups, 0.432	During: NS until 4 h when it was lower in H2, **0.027** After: lower in H2 group, **0.020**	During: oxygen transfer capacity NS between groups, >0.05 After: oxygen transfer capacity better in hydrogen group than control, **0.033**	During: Higher in hydrogen group, **0.031** After: Higher in hydrogen group, **0.002**	–	–	After: Lung injury severity score: 2 or 3 0 or 1, **0.0159**
Hartwig et al,^19^ 2023	Control gNO iNO	1615.6–2255.6 g 1406.9–1604.3 g 3881.3 decreased to (not specified)	–	–	PO_2_ (perfusate): Decrease Increase Increase	–	–	–	Lung injury score: 4.1 to 1.5 4.3 to 2.3 4.0 to 2.3 All NS
Hees et al,^20^ 2025	Control Argon	–	With 10^–5^ mmol/L phenylephrine (% increase in PVR): 136 ± 7% 124 ± 10%, **0.03** With 10^–4^ mmol/L phenylephrine (% increase in PVR): 156 ± 6% 136 ± 17%, **0.02**	With 10^–5^ mmol/L phenylephrine (% increase in PVR): 131 ± 10% 121 ± 8%, **0.02** With 10^–4^ mmol/L phenylephrine (% increase in PVR): 148 ± 15% 132 ± 14%, **0.02**	–	–	Airway pressure (mm Hg): 13.4 ± 2.6 11.9 ± 3.1, 0.38	–	–
Hijiya et al,^21^ 2017	β_2_-adrenergic agonist Control	W/D: 4 h after transplantation: 6.4 ± 0.5 10.2 ± 1.6, **<0.001**	After EVLP: In dynes × s × cm^–5^: 1425.8 ± 319.4 1422.1 ± 415.5, 0.473 4 h after transplantation: 553.4 ± 219.9 884.6 ± 146.6, **0.049**	–	After EVLP: PO_2_ (mm Hg): 492.8 ± 63.6 560.0 ± 61.6, 0.064 4 h after transplantation: 602.4 ± 71.6 95.6 ± 68.4, **<0.001**	After EVLP: In mL/cm H_2_O: 23.1 ± 3.2 24.8 ± 5.9, 0.291 4 h after transplantation: 25.9 ± 3.1 12.1 ± 1.4, **<0.001**	–	–	Histological findings: Control: more severe macroscopic pulmonary edema, perivascular edema, infiltration of inflammatory cells, hemorrhage, and hyaline formation within the alveolar space vs β2-adrenergic agonist; no *P* value
Junior et al,^22^ 2025	Intrapleural Topical cooling + Sevoflurane Intrapleural topical cooling Control	Lung weight (g): 420 ± 58, <0.05 vs intrapleural topical cooling alone, <0.001 vs control 760 ± 309, <0.001 vs control 1514 ± 212	Intrapleural topical cooling + sevoflurane: lower, <0.05 vs intrapleural topical cooling	–	P/F ratio: intrapleural topical cooling + sevoflurane: higher, <0.0001 vs intrapleural topical cooling	Intrapleural topical cooling + sevoflurane: higher, <0.0001 vs intrapleural topical cooling	Peak airway pressure: Intrapleural topical cooling + sevoflurane: lower, <0.0001 vs intrapleural topical cooling	–	–
Kalaf-Mussi et al,^23^ 2024	CO Control	W/D: 6 h of EVLP: CO: lower vs control, 0.027 4 h posttransplant: CO: lower vs control, 0.003	6 h EVLP: CO: lower vs control, 0.007	–	6 h EVLP: P/F ratio: NS between groups, 0.371 4 h posttransplant: CO: higher vs control, 0.032	6 h EVLP: NS between groups, 0.202 4 h posttransplant: CO: higher vs control, 0.024	Peak inspiratory pressure: 6 h EVLP: NS between groups, 0.365 4 h posttransplant: CO: lower vs, control, 0.032	–	Lung injury score: 6 h EVLP: NS between groups, 0.206 4 h posttransplant: NS between groups, 0.643
Kondo et al,^24^ 2015	β_2_-adrenoreceptor agonist Control	W/D: 5.72 ± 0.88 8.78 ± 0.61, 0.0027	β_2_-adrenoreceptor agonist: lower vs control, 0.0068	β_2_-adrenoreceptor agonist: lower vs control, <0.001	PO_2_: NS between groups, 0.12	β_2_-adrenoreceptor agonist: Higher vs control, 0.026	Peak airway pressure: β_2_-adrenoreceptor agonist: lower vs control, <0.001	–	Control: more areas of macroscopic, broad, dark red tissue and edematous changes vs β_2_-adrenoreceptor agonist
Loer et al,^25^ 2000	Perfluorocarbon Control	–	In cm H_2_O/L/min: 116 ± 20 101 ± 18, NS by paired *t* test	In cm H_2_O: 21.3 ± 4.3 19.2 ± 3.8, NS by paired *t* test	PO_2_ (torr): 141 ± 7 145 ± 5, NS by paired *t* test	–	–	–	-
Martens et al,^26^ 2016	Argon Xenon Positive control Negative control	W/D: Argon and xenon: NS vs all Positive control: higher vs negative control, <0.05	Argon and xenon: >0.05 vs all Positive control: higher vs negative control, **<0.05**	–	P/F ratio: Argon and xenon: NS vs all Positive control: lower vs negative control, **<0.05**	–	Peak airway pressure: NS between argon, xenon, and positive control, >0.05 0–4 h: NS between all groups, >0.05 At 5–6 h: Negative control: significantly reduced vs all, **<0.05**	–	Histology: Negative control: Absence of alveolar necrosis vs all, **<0.05** NS between groups in the presence of alveolar macrophages, neutrophils, and bronchial inflammation
Martens et al,^27^ 2017	Argon Control	W/D: 7.5 (7.1–8.1) 7.4 (6.8–7.6), 0.47	In dynes × s × cm^–5^: 732 (655–926) 628 (473–827), 0.24	–	P/F ratio: 370 (325–507) 365 (337–567), 0.79	–	Peak airway pressure (cm H_2_O): 18.5 (15.8–22.8) 19.5 (16.5–24.8), 0.82	–	
Michaelsen et al,^28^ 2022	NO Control	W/D: 5.5 ± 0.4 5.1 ± 0.9, 0.628	In dynes × s × cm^–5^: 260.7 ± 71.8 334.8 ± 138.1, 0.095	–	P/F ratio: 308 ± 37.8 320 ± 39.0, 0.586	In mL/cm H_2_O: 48.2 ± 10.4 39.3 ± 8.0, 0.997	Peak airway pressure (cm H_2_O): 11.5 ± 3.6 12.5 ± 4.3, 0.998	In mmol/L: 6.0 ± 2.1 6.8 ± 2.5, 0.694	Histology: NS in acute lung injury between groups, >0.05
Noda et al,^29^ 2014	Hydrogen Control	–	After EVLP: Hydrogen: lower vs control, **<0.01**	–	After EVLP: Hydrogen: higher vs control, **<0.01** After transplantation: Hydrogen: higher vs control, **<0.05**	Hydrogen: higher vs control, **<0.01**	–	After EVLP: Hydrogen: lower vs control, **<0.01**	–
Noda et al,^11^ 2017	6% O_2_ 40% O_2_ 60% O_2_ 100% O_2_	–	40% O_2_: less vs others, **<0.01**	–	After EVLP: P/F ratio: 6% O_2_: NS vs 100% O_2_, >0.05 40% O_2_ : higher, <0.05 vs 6% O_2_ , **<0.05** vs 100% O_2_ 60% O_2_: higher, **<0.05** vs 6% O_2_, <0.01 vs 100% O_2_ 100% O_2_: lower Posttransplant: 6% O_2_: lowest 40% O_2_ : highest, **<0.05** vs 6% O_2_ 60% O_2_: higher, > 0.05 vs 6% O_2_ 100% O_2_: higher, > 0.05 vs 6% O_2_	After EVLP: 6% O_2_: lowest, NS vs 100% and 60% O_2_, >0.05 40% O_2_ : higher, **<0.05** vs 6% O_2_	–	–	After transplantation: Histological analysis: 6% O_2_: severe edema 40% O_2_: less edema 60% O_2_: less edema 100 O_2_: severe edema
Ohata et al,^30^ 2017	Sham (no ischemia) Warm ischemia Fasudil (+60 min warm ischemia)	W/D None Both fasudil and warm ischemia lower vs Sham, <0.001 Fasudil lower vs warm ischemia, *P* < 0.001	Sham and Fasudil lower vs warm ischemia, *P* < 0.001	–	Oxygen partial pressure: Sham and Fasudil higher vs warm ischemia, *P* <0.001	Sham and Fasudil higher vs warm ischemia, *P* <0.001	–	–	Severe perivascular edema with sporadic hemorrhage was observed in the warm ischemia
Steinkühler et al,^31^ 2024	Control S-EVLP	6.67 (–0.22/8.78) 1.39 (–5.12/1.99), *P* > 0.900	–	<20 mm Hg <20 mm Hg	P/F: 347 (341/415) 437 (420/452), 0.056	S-EVLP higher vs control, **0.003**	–	–	–
Wang et al,^32^ 2018	Control S-EVLP	Weight gain (g): 0.72 ± 0.09 0.52 ± 0.06, **0.044**	NS between groups, 0.15	–	Oxygenation: NS between groups, 0.77	In mL × cm H_2_O^–1^ 90 min: 0.41 ± 0.08 0.70 ± 0.21, **0.015** 120 min: 0.44 ± 0.09 0.70 ± 0.19, **0.001** 150 min: 0.45 ± 0.09 0.69 ± 0.2, **<0.001** 180 min: 0.45 ± 0.09 0.69 ± 0.22, **<0.001**	Peak airway pressure (cm H_2_O): 90 min: 7.83 ± 0.75 6.53 ± 1.04, **0.014** 120 min: 7.48 ± 0.82 6.50 ± 1.11, **0.001** 150 min, 7.27 ± 0.48 6.48 ± 1.3, **<0.001** 180 min, 7.18 ± 0.71 6.75 ± 1.43, **<0.001**	–	Histological Perivascular edema score: 0.58 ± 0.09 0.47 ± 0.07, **0.036**
Wu et al,^33^ 2012	Control FiCO_2_ 5% IR FiCO_2_ 5% IR + hypercapnic acidosis FiCO_2_ 10%	W/D: 4.75 ± 0.17 7.87 ± 0.24, <**0.05** vs control 5.97 ± 1.01, **<0.05** vs IR FiCO_2_ 5%	–	IR FiCO_2_ 5% higher vs control, **<0.05** IR + hypercapnic acidosis FiCO_2_ 10% lower vs IR FiCO_2_ 5%, **<0.05**	Change in PO_2_: final—baseline: –2.7 ± 0.6 –14.3 ± 3.1, **<0.05** vs control –6.0 ± 3.0, **<0.05** vs IR hypercapnic acidosis FiCO_2_ 10%	–	–	–	Histological lung injury score: 0 5, **<0.05** vs control 3, **<0.05** vs IR FiCO_2_ 5%
Yamada et al,^34^ 2017	Control NAC	–	NS between groups, >0.05	–	Oxygenation: NAC higher vs control, 0.053	NS between groups, >0.05	–	–	Based on histology scores for edema: 5.0 ± 0.70 4.50 ± 0.84,0.32
Yamamoto et al,^35^ 2001	Blood-perfused lungs: Alveolar CO_2_ exposure Intravascular CO_2_ exposure Buffer–perfused lungs: Alveolar CO_2_ exposure Intravascular CO_2_ exposure Hypoxia + hypercapnia	-	–	In mm Hg: Blood-perfused lungs: Alveolar CO_2_ exposure: FICO_2_ 0: 13.3 ± 0.7, **<0.05** vs FICO_2_ = 0.06 FICO_2_ 0.03: 15.5 ± 0.5, **<0.05** vs FICO_2_ 0.06 FICO_2_ 0.06: 17.8 ± 1.0 FICO_2_ 0.09: 20.19 ± 1.3, <0.05 vs FICO_2_ 0.06 FICO_2_ 0.12: 23.5 ± 1.9, **<0.05** vs FICO_2_ 0.06 Intravascular CO_2_ exposure: 0% CO_2_: 15.5 ± 0.7, **<0.05** vs 6% CO_2_ 6% CO_2_: 17.0 ± 0.7 12% CO_2_: 18.5 ± 0.8, **<0.05** vs 6% CO_2_ Buffer-perfused lungs: Alveolar CO_2_ exposure: FICO_2_ 0: 12.7 ± 2.3, >0.05 vs FICO_2_ 0.06 FICO_2_ = 0.03: 12.7 ± 2.2, >0.05 vs FICO_2_ 0.06 FICO_2_ 0.06: 13.1 ± 2.3 FICO_2_ = 0.09: 13.6 ± 2.4, >0.05 vs FICO_2_ 0.06 FICO_2_ 0.12: 14.2 ± 2.2, <0.05 vs FICO_2_ 0.06 Intravascular CO_2_ exposure: 0% CO_2_: 13.1 ± 1.0, >0.05 vs 6% CO_2_ 6% CO_2_: 13.2 ± 1.1 12% CO_2_: 13.1 ± 1.1, >0.05 vs 6% CO_2_	–	–	–	–	–

*P*-values are bolded if significant.

ABH, 2-S-amino-6-boronohexanoic acid; CO, carbon monoxide; EVLP, ex vivo lung perfusion; FiCO_2_, fraction of inspired carbon dioxide; FiO_2_, fraction of inspired oxygen; gNO, gaseous nitric oxide; iNO, inhaled nitric oxide gas; IR, ischemia-reperfusion-induced lung injury; NAC, N-acetylcysteine; NO, nitric oxide; NS, no significant difference; P/F ratio, PaO_2_/FiO_2_ ratio; PVR, pulmonary vascular resistance; S-EVLP, sevoflurane ex vivo lung perfusion; W/D, wet-to-dry ratio.

**TABLE 3.** Measures of inflammation, oxidative stress, and apoptosis

Study name	Group	TNFα	IL-1β	IL-6	IL-8	IL-10	Oxidative stress markers:	TUNEL positive cells (%):
Becerra et al,^12^ 2022	Liquid ventilation Control	NS between groups, >0.05	–	NS between groups, >0.05	–	–	–	NS between groups, >0.05
Dong et al,^13^ 2009	NO Control	After EVLP: NO: lower vs control, **0.01** After transplantation: NO: lower vs control, **0.01**	After transplantation: NS between groups, >0.05	After transplantation: NS between groups, >0.05	–	–	–	–
Dong et al,^14^ 2013	CO Control	Posttransplantation: NS between groups, 0.087	Posttransplantation: CO: less vs control, **0.0262**	Posttransplantation: CO: less vs control, **0.0192**	–	–	–	–
Ellman et al,^15^ 2005	21%–24% O_2_ 100% O_2_	–	–	–	–	–	MPO: NS between groups, 0.11	–
George et al,^16^ 2013	Early ABH Late ABH	–	–	–	–	–	Reactive oxygen species: NS between groups, 0.1	–
Haam et al,^17^ 2015	Control Hydrogen	In ng/mL: 0.361 ± 0.132 0.102 ± 0.059, **0.0159**	In ng/mL: 309.7 ± 87.2 193.6 ± 85.5, **0.0317**	In ng/mL: 4.112 ± 1.863 1.524 ± 0.753, **0.0159**	In ng/mL: 40.125 ± 19.382 2.538 ± 1.833, **0.0195**	–	MPO In mU/mg: 51.5 ± 2.3 54.5 ± 1.9, 0.1905	–
Haam et al,^18^ 2018	Control Hydrogen	After transplantation: NS between groups, >0.05	–	After transplantation: Hydrogen: lower vs control group, **0.024**	–	After transplantation: Hydrogen: higher vs, control, **0.037**	–	After transplantation: 3.4 ± 1.6 2.1 ± 0.4, no *P*-value reported
Hartwig et al,^19^ 2023	Control gNO iNO	–	–	–	–	–	–	–
Hees et al,^20^ 2025	Control Argon	–	–	–	–	–	–	–
Hijiya et al,^21^ 2017	β_2_-adrenergic agonist Control	–	–	–	–	–	–	–
Junior et al,^22^ 2025	Intrapleural topical cooling + sevoflurane Intrapleural topical cooling Control	Perfusate: Intrapleural topical cooling + sevoflurane: lower vs intrapleural topical cooling, 0.063 Tissue: NS between groups, >0.05	Perfusate: Intrapleural topical cooling + sevoflurane: lower vs intrapleural topical cooling, 0.0973 Tissue: NS between groups, >0.05	Perfusate: Intrapleural topical cooling + sevoflurane: lower vs intrapleural topical cooling, <0.01 Tissue: NS between groups, >0.05	Perfusate: Intrapleural topical cooling + sevoflurane: lower vs intrapleural topical cooling, <0.05 Tissue: NS between groups, >0.05	Perfusate: Intrapleural topical cooling + sevoflurane: higher vs intrapleural topical cooling, 0.060 Tissue: NS between groups, >0.05	–	NS between intrapleural topical cooling + sevoflurane vs intrapleural topical cooling, >0.05
Kalaf-Mussi et al,^23^ 2024	CO Control	In pg/mL: 1 h EVLP in perfusate: 29.57 (16.16–51.68) 22.53 (18.03–84.67), 0.91 6 h EVLP in perfusate: 192.0 (108.8–372.3) 263.50 (124.6–352.4), 0.52 4 h posttransplant in plasma: 50.5 (29.2–105.4) 43.8 (28.9–54.7), 0.52 In pg/mg protein: 6 h EVLP in tissue: 34.9 (6.78–48.9) 14.1 (0.0–38.2), 0.058 4 h posttransplant in tissue: 10.9 (0.49–80.3) 8.80 (5.04–18.0), 0.91	In pg/mL: 6 h EVLP in perfusate: 26.52 (4.63–56.72) 25.57 (22.84–60.85), 0.56 In pg/mg protein: 1 h EVLP in tissue: 67.9 (29.0–101.4) 38.9 (18.8–83.6), 0.058 6 h EVLP in tissue: 134.9 (29.8–434.3) 68.4 (57.7–118.7), 0.25 4 h posttransplant in tissue: 585.1 (350.0–2430) 443.9 (148.4–1106), 0.34	In pg/mL: 6 h EVLP in perfusate: 567.2 (398.0–1644) 964.9 (364.1–1195), 0.91 4 h posttransplant in plasma: 63.9 (53.4–94.4) 128.9 (77.1–169.5), **0.03** In pg/mg protein: 1 h EVLP in tissue: 12.5 (2.68–33.3) 5.94 (3.08–10.4), 0.17 6 h EVLP in perfusate: 288.4 (59.2–734.9) 99.0 (73.9–228.1), 0.17 4 h posttransplant in tissue: 267.5 (201.1–855.9) 241.9 (28.4–315.6), 0.25	In pg/mL: 6 h EVLP in perfusate: 795.2 (276.7–1374) 1269 (612.3–2063), 0.17 In pg/mg protein: 4 h posttransplant in tissue: 2420 (493–4525) 3123 (875.2–3482), 0.46	In pg/mL: 1 h EVLP in perfusate: 8.27 (5.42–13.02) 7.29 (5.88–7.78), 0.29 6 h EVLP in perfusate: 16.4 (7.78–18.81) 14.14 (12.47–17.61), 0.52 4 h posttransplant in plasma: 9.80 (6.34–11.9) 8.27 (5.42–11.9), 0.67 In pg/mg protein: 1 h EVLP in tissue: 28.7 (25.2–58.4) 25.3 (19.7–26.6), **0.04** 6 h EVLP in tissue: 23.0 (13.2–52.7) 19.4 (15.1–23.5), 0.25 4 h posttransplant in tissue: 32.0 (23.1–37.1) 23.8 (14.8–26.3), **0.04**	MPO In ng/mL: 1 h EVLP in perfusate: 74.6 (41.6–113.6) 43.0 (36.5–51.0), 0.11 6 h EVLP in perfusate: 66.3 (41.6–72.2) 49.5 (47.4–68.7), 0.25 In ng/mg protein: 1 h EVLP in tissue: 257.5 (140.5–327.3) 167.3 (140.5–335.5), 0.46 6 h EVLP in tissue: 147.8 (99.3–213.1) 115.1 (85.9–169.0), 0.46 4 h posttransplant in tissue: 207.4 (114.4–237.9) 532.5 (298.7–893.5), **0.009**	6 h EVLP: 0.3226 ± 0.3221 1.408 ± 1.409, 0.222 4 h posttransplant: 0.4151 ± 0.3397 1.962 ± 2.164, 0.151
Kondo et al,^24^ 2015	β_2_-adrenoreceptor agonist Control	–	–	–	NS between groups, *P* > 0.05	–	–	–
Loer et al,^25^ 2000	Perfluorocarbon Control	–	–	–	–	–	–	–
Martens et al,^26^ 2016	Argon Xenon Positive control Negative control	–	–	–	–	–	–	–
Martens et al,^27^ 2017	Argon Control	Bronchoalveolar lavage: 61 (20–243) 0.03 (0.03–69), 0.11 In perfusate: 833 (5664–7477) 322 (254–1067), 0.24	–	Bronchoalveolar lavage: 525 (214–1730) 152 (123–807), 0.24 In perfusate: 2451 (697–4818) 904 (386–127.6), 0.09	–	–	–	–
Michaelsen et al,^28^ 2022	NO Control	–	In pg/mL: In perfusate: 1 h: 1.3 (0.5–3.8) 0.1 (0.0–0.4), 0.285 6 h: 1.1 (0.2–4.5) 0.3 (0.0–0.9), 0.485 12 h: 7.1 (3.2–13.5) 2.5 (0.8–9.2), 0.685 In tissue: Pre-EVLP: 61.3 (55.5–104.6) 100.4 (94.8–101.5), 0.628 Post-EVLP: 96.1 (92.8–108.3) 98.4 (90.2–114.3), 0.685	In pg/mL: In perfusate: 1 h: 5.9 (5.2–7.9) 8.6 (6.5–22.9), 0.485 6 h: 226.8 (127.5–310.3) 192.8 (180.1–229.4), >0.999 12 h: 1114.6 (932.3–1247) 626.9 (601.4–664.8), 0.342 In tissue: Pre-EVLP: 33.9 (31.2–34) 31.9 (29.5–35.4), 0.857 Post-EVLP: 155.6 (95.1–272.1) 71.4 (41.3–163.8), 0.342	In pg/mL: In perfusate: 1 h: 44.7 (44.3–45.1) 44.7 (42.6–47.6), >0.999 6 h: 68.8 (63.0–84.3) 54.7 (48.0–60.5), 0.200 12 h: 110.5 (96.8–158.8) 73.8 (67.6–87.2), 0.485 In tissue: Pre-EVLP: 38.3 (36.3–39.0) 32.4 (30.3–33.8), 0.114 Post-EVLP: 167.4 (121.5–294.4) 111.5 (51.4–187.8), 0.685	–	–	–
Noda et al,^29^ 2014	Hydrogen Control	After EVLP: Hydrogen: lower vs control, **<0.01** After transplantation: Hydrogen: lower vs control, **<0.05**	After EVLP: Hydrogen: lower vs control, **<0.01** After transplantation: Hydrogen: lower vs control, **<0.01**	After EVLP: Hydrogen: lower vs control, **<0.01** After transplantation: Hydrogen: lower vs control, **<0.05**	–	–	–	–
Noda et al,^11^ 2017	6% O_2_ 40% O_2_ 60% O_2_ 100% O_2_	After EVLP: 6% O_2_: NS vs 100% O_2_, >0.05 40% O_2_: lower, **<0.01** vs 6% O_2_, **<0.01** vs 100% O_2_ 60% O_2_: lower, **<0.01** vs 6% O_2_, **<0.01** vs 100% O_2_ 100% O_2_: higher Posttransplant: 6% O_2_: lower vs 100% O_2_ <**0.01** 40% O_2_: lower**, <0.01** vs 6% O_2_ and **<0.01** vs 100% O_2_ 60% O_2_: lower, **<0.01** vs 6% O_2_, **<0.01** vs 100% O_2_ 100% O_2_: highest	After EVLP: 6% O_2_: NS vs 100% O_2_, >0.05 40% O_2_: lower, **<0.01** vs 6% O_2_, **<0.01** vs 100% O_2_ 60% O_2_: lower, **<0.01** vs 6% O_2_, **<0.01** vs 100% O_2_ 100% O_2_: higher Posttransplant: 6% O_2_: highest 40% O_2_: lower, **<0.01** vs 6% O_2_, >0.05 vs 100% O_2_ 60% O_2_: NS vs 6% O_2_, >0.05 100% O_2_: lower vs 6% O_2_, **<0.01**	After EVLP: 6% O_2_: NS vs 100% O_2_, >0.05 40% O_2_: lower, **<0.01** vs 6% O_2_, **<0.01** vs 100% O_2_ 60% O_2_: lower, **<0.01** vs 6% O_2_, **<0.01** vs 100% O_2_ 100% O_2_: higher Posttransplant: 6% O_2_: highest 40% O_2_: lower, **<0.01** vs 6% O_2_ 60% O_2_: lower, **<0.01** vs 6% O_2_ 100% O_2_: lower, **<0.01** vs 6% O_2_	–	–	MDA levels: higher in 100% vs all, **<0.05**	After EVLP: 6% O_2_: NS vs 100% O_2_, >0.05 40% O_2_: lower, **<0.01** vs 6% O_2_, **<0.01** vs 100% O_2_ 60% O_2_: lower, **<0.01** vs 6% O_2_, **<0.01** vs 100% O_2_ 100% O_2_: higher
Ohata et al,^30^ 2017	Sham (no ischemia) Warm ischemia Fasudil (+60 min warm ischemia)	–	–	–	–	–	–	–
Steinkühler et al,^31^ 2024	Control S-EVLP	NS between groups, >0.05	–	Post-pre EVLP: 3.385 (0.449–6.102) 0.708 (0.394–5.751), 0.691	–	–	MPO Post-pre EVLP: 0.0004 (–0.0443 to 0.0479), –0.0198 (–0.3413 to –0.0028), 0.222	–
Wang et al,^32^ 2018	Control S-EVLP	In ng/mg: 1.50 ± 0.59 0.59 ± 0.38, **0.02**	–	In ng/mg: 2.63 ± 1.06 1.58 ± 0.29, 0.11	In ng/mg: 1.17 ± 0.32 0.66 ± 0.28, **0.03**		Protein carbonyl (nmol/mg): 1.17 ± 0.44 0.55 ± 0.11, **0.006**	
Wu et al,^33^ 2012	Control: FiCO_2_ 5% IR FiCO_2_ 5% IR + hypercapnic acidosis FiCO_2_ 10%	IR FiCO_2_ 5%: higher vs control and HCA, **<0.05** NS between hypercapnic acidosis and control, >0.05	–	–	–	IR FiCO_2_ 5%: higher vs control and hypercapnic acidosis, **<0.05**	MDA levels (nmol/mg protein): 4.95 ± 0.26 5.81 ± 0.56, **<0.05** vs control 4.98 ± 0.44, **<0.05** vs IR FiCO_2_ 5%	–
Yamada et al,^34^ 2017	Control NAC	–	NS between groups, 0.18	–	In pg/mL: 874 ± 544 429 ± 162, 0.09	–	MPO In U/mL: 34.2 ± 5.4 25.4 ± 6.4, **0.037** MDA and protein carbonylation: NS between groups for protein carbonylation (*P* = 0.13) and MDA (*P* = 0.17)	–

*P*-values are bolded if significant. Dashes indicates that data were not available for the indicated variable.

ABH, 2-S-amino-6-boronohexanoic acid; CO, carbon monoxide; EVLP, ex vivo lung perfusion; FiCO_2_, fraction of inspired carbon dioxide; FiO_2_, fraction of inspired oxygen; gNO, gaseous nitric oxide; HCA, hypercapnic acidosis; IL, interleukin; iNO, inhaled nitric oxide gas; IR, ischemia-reperfusion-induced lung injury; MDA, malondialdehyde; MPO, myeloperoxidase; NAC, N-acetylcysteine; NO, nitric oxide; NS, no significant difference; S-EVLP, sevoflurane ex vivo lung perfusion; TNFα, tumor necrosis factor alpha; TUNEL+, terminal deoxynucleotidyl transferase-mediated dUTP nick-end labeling positive cells.

A detailed results description is provided in the **Supplemental Materials (SDC**, https://links.lww.com/TXD/A907), with key findings summarized in the subsequent sections.

### Lung and Airway Function

Lung and airway function were measured using various parameters, which consisted of peak airway pressure (PAW), peak inspiratory pressure (PIP), oxygenation (PO_2_ and PaO_2_/FiO_2_ [P/F] ratio), PVR, PAP, and compliance. In terms of other measures of lung integrity, increased PAP and PVR, while decreased oxygenation, indicated poor lung function.

#### Vasoactive Gases (NO, CO, CO_2_, O_2_)

Nine studies evaluated vasoactive gases, which are produced endogenously and affect pulmonary circulation. PVR was reduced by CO and 40% O_2_ but showed no significant change with NO.

In the study by Kalaf-Mussi et al,^23^ the addition of 500 ppm CO for the first hour of EVLP to the gas mixture containing 8% CO_2_, 6% O_2_, and balanced nitrogen (N_2_), which ran for 6 h total, followed by 4 h of observation after left lung transplantation, resulted in decreased PVR after EVLP but did not affect P/F ratio, PIP, and compliance after EVLP. However, increases in P/F ratio and compliance, while a decrease in PIP, occurred 4 h after transplantation.^23^ In the study by Noda et al,^11^ varying O_2_ concentrations (6%–100%), only 40% O_2_ decreased PVR and increased compliance compared with 6% O_2_. However, Po_2_ increased with 40% and 60% O_2_ compared with 6% and 100% O_2_.^11^ Ellman et al^15^ compared 21%–24% O_2_ to 100% O_2_ in 3-h EVLP. Results showed no significant differences in PAP, P/F ratio, or PIP.^15^

Administration of 200 ppm NO with 86% N_2_, 8% CO_2_, and 6% O_2_ during 12-h porcine EVLP did not result in any significant changes to PVR, P/F ratio, compliance, and PAW.^28^ Oxygenation levels were shown to improve when using 80 ppm NO and 20 ppm NO, but statistical significance was not measured.^19^ The P/F ratio improved significantly and mean airway pressure nonsignificantly decreased in rats who received 40 ppm NO and 60% O_2_ compared with 60% O_2_ alone.^13^ Similarly, CO with 60% O_2_ increased P/F ratio and had a nonsignificant decrease in PAW.^14^

CO_2_ administration produced variable effects depending on perfusate composition. In 1 study using acellular perfusate, 10% inspired CO_2_ reduced PAP and lessened oxygen decline compared with 5% inspired CO_2_.^33^ In blood-perfused lungs, 0% and 3% inhaled CO_2_ decreased PAP, whereas 9% and 12% inhaled CO_2_ increased it relative to 6% inhaled CO_2_ control.^35^ The study of acellular buffer-perfused lungs showed that 12% inhaled CO_2_ caused PAP elevation, but 0%, 3%, and 9% inhaled CO_2_ concentrations did not affect PAP.^35^

#### Inert and Cytoprotective Gases (Ar, Xe, H_2_)

Inert and cytoprotective gases such as Ar, Xe, and H_2_ were explored in 6 studies. In porcine 4–6 h EVLP, Ar or Xe showed no significant differences in PVR, PAP, or P/F ratio compared with control groups.^26,27^ PAW remained unchanged in both studies.^26,27^ In a rodent 70-min EVLP, Ar resulted in lower PVR and PAP compared with N_2_ controls.^20^ However, this reduction was not associated with changes in airway pressure.^20^ H_2_ produced more consistent improvements across studies. In porcine models, H_2_ treatment decreased PVR and PAP during EVLP.^17^ Oxygen transfer capacity was not significantly different between groups.^17^ In a follow-up study, H_2_ did not alter PVR during EVLP but improved PAP, compliance, and oxygen transfer capacity after transplantation.^18^ In a rodent model, H_2_ treatment decreased PVR, increased oxygenation, and increased compliance during EVLP and after transplantation.^29^

#### Inhaled Pharmacologic Therapies

Inhaled pharmacologic interventions were evaluated in 5 studies. In a 4-h porcine EVLP, treatment with a β_2_-adrenoreceptor agonist decreased PVR and PAP, increased compliance, and lowered PAW compared with controls, while oxygenation remained unchanged in 1 study and improved in another.^24^ In a subsequent study using the same intervention, PVR and compliance did not differ during 2 h of EVLP, but both parameters, along with oxygenation, improved after transplantation.^21^

Nebulized fasudil also produced favorable changes in vascular and airway function. PVR decreased, and compliance and oxygenation increased compared with controls.^30^ In contrast, NAC did not significantly affect PVR, compliance, and oxygenation.^34^ In human EVLP, ABH did not change PAP but transiently increased dynamic compliance, while oxygenation remained unchanged.^16^

#### Anesthetics and Liquid Ventilation

Sevoflurane and liquid ventilation strategies using perfluorocarbon-based agents were explored in 5 studies. In rat EVLP, administration of sevoflurane with 6% O_2_ improved dynamic compliance and lowered PAW, while PVR and oxygenation did not significantly change.^32^ In porcine EVLP, sevoflurane ventilation with 40% O_2_ increased compliance without significant changes in oxygenation.^31^ In a porcine uncontrolled donation-after-cardiac-death model, intrapleural topical cooling and 50% O_2_ combined with sevoflurane, provided before EVLP, reduced PVR and PAW, while compliance and P/F ratio were significantly higher compared with intrapleural topical cooling alone^22^

Liquid ventilation with perfluorocarbon did not significantly alter PVR, PAP, and oxygenation.^25^ Similarly, total liquid ventilation during EVLP after ischemia-reperfusion injury did not significantly affect PVR, compliance, or partial pressure of O_2_ compared with controls.^12^

### Inflammatory, Oxidative Stress, and Apoptosis Markers

Inflammatory markers included tumor necrosis factor (TNF)-α, pro-inflammatory interleukin (IL)-6, IL-1β, IL-8, and anti-inflammatory IL-10, with elevated levels indicating increased inflammation. Oxidative stress was measured using malondialdehyde (MDA), myeloperoxidase (MPO), and/or protein carbonyl levels. Terminal deoxynucleotidyl transferase-mediated dUTP nick-end labeling (TUNEL) positive stained cells, with increased amounts indicating increased apoptosis.

#### Vasoactive Gases (NO, CO, CO_2_, O_2_)

Out of the vasoactive gases, NO decreased TNFα in 1 study,^14^ did not affect IL-1β in 2 studies,^13,28^ had no effect on IL-6 in 2 studies,^13,28^ and had no effect on IL-8 in 1 study.^28^ CO, on the other hand, did not affect TNFα in 2 studies,^14,23^ decreased IL-1β when CO was combined with 60% O_2_ in 1 study,^14^ had no effect when CO was provided with 6% O_2_ in another study,^23^ decreased IL-6 in 2 studies,^14,23^ did not affect IL-8 in 1 study,^23^ increased IL-10 in tissue but not perfusate in 1 study,^23^ reduced MPO after transplant but not during EVLP in 1 study,^23^ and had no effect on TUNEL+ cells.^23^

Effect of O_2_ varied by concentration. In 1 study, 40% and 60% O_2_ consistently decreased TNFα, IL-1β, and IL-6 after EVLP, but IL-1β and IL-6 remained lower after transplant only with 40% O_2_, whereas TNFα remained lower after transplant and reduced TUNEL+ cells in both 40% and 60% O_2_ compared with 6% and 100% O_2_.^11^ Ellman et al^15^ additionally did not find any difference in MPO between 21% and 24% O_2_ compared with 100% O_2_. Decreased inflammation and oxidative stress from CO_2_ also differed by concentration, with 1 study showing decreased TNFα, IL-10, and MDA in 10% CO_2_ compared with 5% CO_2_.^33^

#### Inert and Cytoprotective Gases (Ar, Xe, H_2_)

Ar did not change TNFα or IL-6 levels compared with controls in 1 study.^27^ H_2_ produced greater anti-inflammatory effects. H_2_ reduced TNFα, IL-1β, IL-6, and IL-8 production but did not affect MPO after EVLP compared with the control in 1 study.^17^ However, the effect of H_2_ after transplantation following EVLP was found to reduce IL-6 and increase IL-10, but did not affect TNFα in another study.^18^ In another study that used a shorter duration of EVLP before transplantation, TNFα, IL-6, and IL-1β were reduced posttransplantation in H_2_-treated lungs compared with the control group.^29^ The TUNEL staining results showed that H_2_ treatment decreased apoptotic cells in lung tissue to 2.1 ± 0.4 compared with 3.4 ± 1.6 in control samples, but the study did not provide a *P*-value.^18^

#### Inhaled Pharmacologic Therapies

Administration of a β_2_-adrenoreceptor agonist did not significantly alter IL-8 levels compared with controls in 1 study.^24^ NAC treatment showed no difference in IL-1β and IL-8 compared with control lungs in 1 study.^34^ For oxidative markers, although NAC treatment demonstrated no change in MDA and protein carbonylation, there was a decrease in MPO activity compared with the control in 1 study.^34^ One study showed that nebulized ABH treatment did not produce any significant change in reactive oxygen species (ROS) production.^16^

#### Anesthetics and Liquid Ventilation

Sevoflurane with 6% O_2_ was found to reduce TNFα, IL-8, and protein carbonylation, but did not affect IL-6 in 1 study.^32^ However, sevoflurane provided either before or during EVLP, with 40% or 50% O_2,_ was found not to affect TNFα in 2 studies,^22,31^ had no effect on IL-1β in 1 study,^22^ decreased IL-8 in 1 study,^22^ increased IL-10 in 1 study,^22^ and had no effect on TUNEL+ cells in 1 study.^31^ IL-6 was decreased when sevoflurane and 50% O_2_ were added with intrapleural topical cooling compared with intrapleural topical cooling alone in 1 study.^22^ No change in IL-6 was seen when sevoflurane and 40% O_2_ were compared with 40% O_2_ alone in another study.^31^ Liquid ventilation compared with gas ventilation produced no significant differences in TNFα, IL-6, or TUNEL staining in 1 study.^12^

### Edema, Metabolic Homeostasis, and Histological Lung Injury Score

Lung injury was assessed by reporting increased edema formation, lactate, glucose consumption, and histological lung injury score. Edema formation consisted of either wet-to-dry lung weight ratio (W/D) or weight gain.

#### Vasoactive Gases (NO, CO, CO_2_, O_2_)

CO compared with control was found to reduce the W/D ratio in 2 studies,^14,23^ but had no significant effect on histological lung injury score in 1 study.^23^ In contrast, NO effects varied by concentration and concurrent O_2_ concentration. 60% O_2_ and 40 ppm NO reduced the W/D ratio after EVLP but showed no difference following transplantation in 1 study,^13^ whereas 200 ppm NO exposure with a gas mixture containing 6% O_2_ did not significantly affect W/D ratio, lactate levels, and histological lung injury score during EVLP in another study.^28^ A follow-up clinical pilot demonstrated that 20 ppm NO or 80 ppm NO with 6% O_2_, compared with 6% O_2_ alone, lowered lung weight after 6-h EVLP, but had minimal effect on histological injury score, although significance was not measured.^19^

Ten percent CO_2_, compared with 5% CO_2_, also decreased lung weight-to-body weight ratio and improved histological injury scores in 1 study.^33^ In another study, exposure to 21%–24% O_2_ produced lower W/D ratios compared with 100% O_2_.^15^ Finally, lungs perfused with 40% and 60% O_2_ showed less edema on histological analysis compared with 6% and 100% O_2_ groups; however, statistical significance was not measured.^11^

#### Inert and Cytoprotective Gases (Ar, Xe, H_2_)

Ar did not alter the W/D ratio in 2 studies,^26,27^ and Ar and Xe did not alter histological score in terms of alveolar macrophages, neutrophils, alveolar necrosis, and bronchial inflammation in 1 study.^26^ H_2_ significantly lowered the W/D ratio after EVLP^17^ in 1 study, did not have significant effects on W/D ratio after transplant in another study,^18^ lowered histologic lung injury scores in 2 studies,^17,18^ and decreased lactate in 1 study.^29^

#### Inhaled Pharmacologic Therapies

Treatment with a β_2_-adrenoreceptor agonist decreased the W/D ratio and improved histologic architecture, in terms of macroscopic lung appearance and presence of edematous tissue, in 2 studies.^21,24^ Nebulized fasudil compared with the control also decreased the W/D ratio and yielded improved histologic preservation, described by attenuated edema and hemorrhage, in 1 study.^30^ By contrast, NAC treatment during porcine EVLP led to stable histologic scores without significant differences from control lungs.^34^

#### Anesthetics and Liquid Ventilation

Lungs ventilated with sevoflurane before or during EVLP demonstrated reduced edema in 2 studies^22,32^ and reduced histologic lung injury score in 1 study.^32^ Liquid ventilation showed no significant changes in W/D ratio and lactate in 1 study.^12^

## DISCUSSION

**FIGURE 3.**
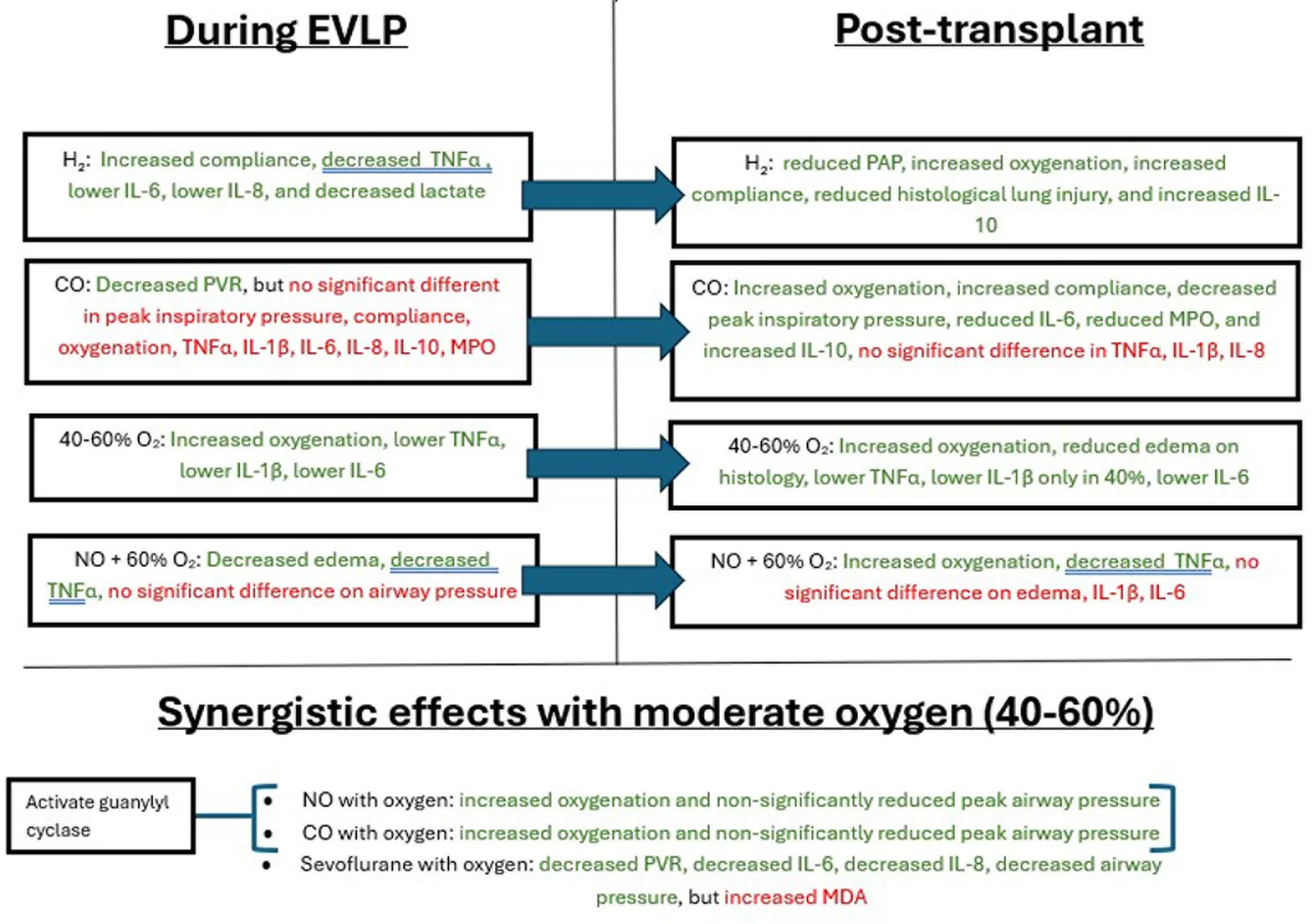
Heatmap illustrating the use and effects of various inhaled agents during EVLP. CO, carbon monoxide; EVLP, ex vivo lung perfusion; H_2_, hydrogen; IL, interleukin; MDA, malondialdehyde; MPO, myeloperoxidase; NO, nitric oxide; O_2_, oxygen; PAP, pulmonary artery pressure; PVR, pulmonary vascular resistance; TNFα, tumor necrosis factor alpha.

This review examined inhaled and gaseous interventions during EVLP and subsequent transplantation (Figure 3). Notably, the beneficial effects of H_2_ and CO were more evident after transplantation following EVLP rather than during EVLP itself, suggesting delayed or sustained protective mechanisms. Additional synergistic effects were observed when 40%–60% O_2_ was combined with either CO, NO, or sevoflurane compared with O_2_ alone. Minimal benefits were seen when NO or sevoflurane with 6% O_2_, liquid ventilation, Ar, Xe, and NAC nebulization were provided.

Responses to CO_2_ varied by perfusate composition. In blood-perfused circuits, CO_2_ concentrations >9% increased PAP, whereas acellular perfusates did not experience an increase in PAP until 12% CO_2._ This aligns with Wu et al,^33^ who reported benefits at 10% CO_2_ using an acellular, salt-based perfusate. The adverse effects of increased CO_2_ in blood-perfused circuits may relate to the Bohr-mediated acidosis from red-cell carbonic anhydrase, leading to pulmonary vasoconstriction in the poorly buffered isolated EVLP circuit.^36^ However, CO_2_ can also cause pH-independent vasodilation under high vascular tone.^36^ This dual behavior may explain the consistent improvements observed with 10% versus 5% CO_2_ in acellular perfusates.

CO exposure, on the other hand, consistently reduced PVR and edema, while increasing compliance, oxygenation, and IL-10. These improvements align with existing literature describing the multifactorial effects of CO, including its antioxidant, anti-inflammatory, and organ-protective properties mediated through binding to various proteins such as hemoglobin, myoglobin, and cytoglobin.^37^ While additional reductions in IL-1β, IL-6, MPO, and airway pressure observed after transplantation in our review suggest that the primary effects of CO on airway function and inflammation may manifest during reperfusion rather than during EVLP. Of note, MPO, used as a marker of oxidative stress in our review, is an enzyme released from neutrophils under inflammatory and oxidative stress conditions; its reduction posttransplantation is consistent with attenuated leukocyte recruitment.^38^ Hence, while these protective effects of CO may be initiated during EVLP, their magnitude may not be significant compared with controls until later time points.

Like CO, H_2_ showed more protective effects following transplantation rather than during EVLP. After EVLP, H_2_ decreased TNFα, IL-1β, IL-8, and edema, while following transplantation, it further decreased PAP and increased compliance, IL-10, and oxygenation. Histological lung injury scores were also reduced both after EVLP and posttransplantation. H_2_ treatment has been shown to inhibit pulmonary cell apoptosis and suppress both early and late nuclear factor κB-mediated inflammation, consistent with the early reductions in TNFα, IL-1β, IL-8, and the later rise in IL-10.^39,40^ Additionally, H_2_ has been reported to upregulate tight junction proteins, thereby maintaining the integrity of the lung epithelial barrier and potentially explaining the improved histological findings.^41^ The more pronounced benefits seen after transplantation than during EVLP may reflect the fact that H_2_ and CO primarily preserve lung architecture and attenuate subclinical injury during EVLP, effects that may not immediately translate into physiological improvements. However, with CO mainly reducing airway remodeling and H_2_ maintaining lung epithelium, combining CO and H_2_ during EVLP may provide protection of the whole lung, offering further improvement in graft function.

Across studies manipulating oxygen concentration, 40%–60% O_2_ reduced TNFα, IL-6, and apoptosis, demonstrating a balance between avoiding both hyperoxic oxidative injury and hypoxia-induced pulmonary vasoconstriction. These findings are consistent with existing evidence suggesting that 40%–60% O_2_ minimized mitochondrial impairment, dampened nuclear factor κB activation, and downregulated lung septation and angiogenesis, which ultimately reduce inflammatory signaling and prevent cell death.^42-44^ Our review further showed that 100% O_2_ increased MDA levels but had no effect on MPO compared with other oxygen concentrations. MDA is a reactive aldehyde formed through lipid peroxidation in the presence of ROS.^38,45^ Hence, this suggests that high oxygen concentrations increase ROS, leading to oxidative damage. Interestingly, this observation aligns with Ruigrok et al,^44^ who reported increased ROS production through DNA fragmentation and upregulation of antioxidant target genes in lung tissue exposed to 80% O_2_. Conversely, very low oxygen concentrations, which were observed at 6% O_2_ in our review, may impair cellular metabolism and trigger hypoxic pulmonary vasoconstriction, increasing PVR and impairing perfusion distribution.

NO demonstrated dose- and oxygen-dependent effects. Administration of 40 ppm NO with 60% O_2_ compared with O_2_ alone improved oxygenation, airway pressure, TNFα, and edema, whereas 200 ppm NO with 6% O_2_ had no significant effects. The findings highlight a potential synergistic effect between NO and 60% O_2_ concentration, as well as the potential for excessive NO to blunt potential benefits. However, further studies exploring different NO and O_2_ combinations are warranted.

Similar synergistic effects were seen with CO, with 60% O_2_ improved oxygenation and reduced edema, IL-1β, and IL-6 compared with 60% O_2_ alone. The parallels between CO and NO may be attributable to their shared vasodilatory effects through activation of guanylate cyclase.^46^ Vasodilation may have aided oxygen delivery, thereby amplifying the beneficial impact of 60% O_2_.

Similarly, sevoflurane with 50% O_2_ compared with O_2_ alone decreased PVR, IL-6, IL-8, and airway pressure, while increasing compliance and IL-10. Interestingly, 2% sevoflurane with 6% O_2_ compared with 40% or 50% O_2_ further reduced TNFα and protein carbonyl levels. Protein carbonyl levels, similar to MDA, reflect the increased presence of ROS.^47^ These findings suggest that sevoflurane may exert optimal anti-inflammatory, vascular, and airway-protective effects at <50% O_2_, although this O_2_ concentration may exacerbate oxidative stress in bronchodilated lungs.

Among inhaled medications, both β_2_-adrenoreceptor agonists and fasudil improved compliance and oxygenation while decreasing PVR, histological lung injury scores, and edema. ABH similarly demonstrated anti-inflammatory and vasodilatory properties by inhibiting the breakdown of L-arginine, thereby increasing substrate availability for NO synthase and promoting endogenous NO production.^46^ Comparable to NO, ABH reduced edema; however, unlike NO, it did not improve oxygenation. It is possible that coadministration of 60% O_2_ with ABH, as seen with NO, could enhance oxygenation. Additional effects of ABH included increased compliance, although no significant changes were observed in PAP or oxidative stress.

Collectively, these findings suggest that combining complementary interventions may yield more comprehensive protection and improve lung viability during and after EVLP. Among the interventions evaluated, NO or sevoflurane combined with 40%–60% O_2_ may be the most clinically translatable because both agents are already routinely used in critical care settings with established guidelines. In contrast, H_2_ and CO are currently undergoing clinical trials for optimal dosing and usage in additional clinical settings, which may limit implementation despite promising experimental results. Additionally, these findings should be interpreted in the context of the available evidence base, as most studies included in this review were conducted on animal models, with most on porcine and rodents. While porcine lungs share many physiological and anatomical characteristics with human lungs, including comparable organ size, airway branching patterns, pulmonary vascular architecture, and immune responses, rodent models differ from human lungs, which may limit clinical translation.^48^ Furthermore, many experimental models evaluate short-term physiological outcomes rather than long-term clinical outcomes such as PGD, long-term graft function, and recipient survival. An additional consideration is that many donor lungs have likely already experienced mechanical injury, inflammation, aspiration, or edema before procurement. Future research should prioritize using combined inhaled interventions in large-animal models and, ultimately, human clinical studies to determine their safety, ability to reverse previous donor lung injury, and impact on posttransplant outcomes.

### Limitations

The current review is constrained by variability in gas and inhaled intervention protocols, small sample sizes, and brief EVLP or posttransplant follow-up periods, which limited the generalizability of the results. Finally, the potential for publication bias cannot be excluded, as studies with neutral or negative results may be underrepresented, possibly exaggerating the perceived efficacy in some studies.

## CONCLUSIONS

This review demonstrated that inhaled interventions during EVLP and subsequent transplantation exert variable effects on lung function, inflammation, oxidative stress, and posttransplantation lung function. While most interventions assessed offered improved lung function compared with control treatment during EVLP, the protective effects of CO and H_2_ were more pronounced after transplantation following EVLP, suggesting more sustained or delayed effects from these interventions. Additionally, NO, sevoflurane, and CO in combination with 50%–60% O_2_ had additional synergistic effects compared with each therapy alone. Future studies should focus on elucidating the mechanism of these agents, defining optimal treatment regimens, and investigating long-term impacts on posttransplant recovery.

## Supplementary Material


