# Supplementary material for: Comparing Gas and Inhaled Pharmacologic Interventions During Ex Vivo Lung Perfusion: A Systematic Review
Source: Transplant Direct. 2026 Sep 16;12(10):e2012. doi: 10.1097/TXD.0000000000002012 (PMC13593184; doi:10.1097/TXD.0000000000002012)
Supplement: Supplementary file 1 [file txd-12-e2012-s001.pdf]

Comparing Gas and Inhaled Pharmacologic Interventions During Ex Vivo Lung Perfusion A Systematic Review

Supplemental Materials

3.2 Lung and Airway Function

Lung and airway function were measured using various parameters, which consisted of peak airway pressure (PAW), peak inspiratory pressure (PIP), oxygenation (partial pressure of oxygen (PO_2) and PaO_2/FiO_2 or P/F ratio), pulmonary vascular resistance (PVR), pulmonary artery pressure (PAP), and compliance. In terms of other measures of lung integrity, increased PAP and PVR, while decreased oxygenation indicated poor lung function.

3.2.1 Vasoactive Gases (NO, CO, CO_2, O_2)

Nine studies evaluated vasoactive gases, which are produced endogenously and affect pulmonary circulation. PVR was reduced by carbon monoxide (CO) in one study[1], 40% oxygen (O_2) in one study[2], but had no significant effect when nitric oxide (NO) was administered in one study [3]. Kalaf-Mussi et al.[1] evaluated 500 ppm CO for the first hour of EVLP to the gas mixture containing 8% CO_2, 6% O_2, and balanced nitrogen (N_2) which ran for six hours total, followed by four hours of observation after left lung transplantation in a porcine model. The addition of CO during compared to controls led to decreased PVR after EVLP ($p=0.007$), no significant difference in P/F ratio and compliance after EVLP ($p=0.371$ and $p=0.202$, respectively) but a significant increase 4 hours after transplantation ($p=0.032$ and $p=0.024$, respectively)[1]. Similarly, PIP was not significantly different after EVLP but decreased after transplantation in CO-treated lung compared to controls ($p=0.365$ and $p=0.032$, respectively) [1]. Noda et al. [2] evaluated various O_2

concentrations (6-100%) with 8% CO_2 and balanced N_2 during 4 hours of EVLP in a rat model. They found that PVR was only decreased when 40% O_2 was used compared to the other concentrations ($p<0.01$), 40% and 60% O_2 increased partial pressure of O_2 compared to 6% and 100% O_2 ($p<0.05$ and $p<0.01$, respectively), and compliance increased only with 40% O_2 compared to 6% O_2 ($p<0.05$), whereas the 60% and 100% O_2 had no significant difference compared to 6% O_2 ($p>0.05$)[2]. 21-24% O_2 was compared to 100% O_2 during three hours of EVLP in a rabbit model in Ellman et al.[4]. There were no significant differences in PAP, P/F ratio, and PIP between 21-24% O_2 and 100% O_2 ($p>0.05$ for all)[4].

Michaelsen et al.[3] assessed the administration of 200 ppm NO with 86% N_2, 8% CO_2, and 6% O_2 during 12 hours of EVLP in a porcine model. They reported no significant difference in PVR (260.7 ± 71.8 dynes×sec×cm^{-5} vs. 334.8 ± 138.1 dynes×sec×cm^{-5}, p=0.095), P/F ratio (308 ± 37.8 vs. 320 ± 39.0, p=0.586), compliance (48.2 ± 10.4 mL/cmH_2O vs. 39.3 ± 8.0 mL/cmH_2O, p=0.997), and PAW (11.5 ± 3.6 cmH_2O vs. 12.5 ± 4.3 cmH_2O, p=0.998) in NO-treated compared to control group[3]. Other studies that utilized NO were Hartwig et al.[5] and Dong et al. 2009[6]. Hartwig et al.[5] compared administration of 80 ppm NO in gaseous form into the EVLP oxygenator versus 20 ppm NO into the ventilation system, both were added in addition to 6% O_2, 8% CO_2, and 86% N_2 during 6 hours of EVLP. They described that both forms of NO increased partial pressure of O_2 compared to the control group, however, statistical significance and quantitative data were not calculated[5]. Dong et al. 2009[6] also used 40 ppm NO, however, at a concentration of 40 ppm and with 60% O_2 during 1 hour of EVLP, followed by observation of 1 hour post left lung transplantation. NO administration with 60% O_2 compared to 60% O_2 alone increased P/F ratio and a non-significant decrease in mean airway pressure ($p<0.001$ and $p=0.07$, respectively)[6]. In a follow-up study, CO with 60% O_2 in a similar 1-hour EVLP and 1-hour post-

transplantation rat model was used[7]. They similarly found increased P/F ratio and a non-significant decrease in mean airway pressure with CO and 60% O_2 compared to 60% O_2 alone ($p=0.0058$ and $p=0.14$, respectively)[7].

In terms of CO_2 administration, differences were seen depending on whether lungs were perfused with acellular versus blood-containing perfusates during EVLP. Yamamoto et al.[8] assessed the administration of various concentrations of CO_2 (0-12%) with 20% O_2 and balanced N_2 administered either through inhalation or into the perfusate circuit during the first 15 minutes of EVLP. When CO_2 was inhaled in blood perfused lungs, 0% and 3% CO_2 decreased PAP while 9% and 12% CO_2 increased PAP compared to 6% CO_2 control (13.3 ± 0.7 mmHg vs. 17.8 ± 1.0 mmHg, $p<0.05$; 15.5 ± 0.5 mmHg vs. 17.8 ± 1.0 mmHg, $p<0.05$; 20.19 ± 1.3 mmHg vs. 17.8 ± 1.0 mmHg, $p<0.05$; 23.5 ± 1.9 mmHg vs. 17.8 ± 1.0 mmHg, $p<0.05$, respectively) [8]. When CO_2 was inhaled in acellular buffer-perfused lungs, PAP was increased with 12% CO_2, however no significant difference in PAP was seen when 0%, 3%, and 9% CO_2 were provided compared to 6% CO_2 control (14.2 ± 2.2 mmHg vs. 13.1 ± 2.3 mmHg, $p<0.05$; 12.7± 2.3 mmHg vs. 13.1 ± 2.3 mmHg, $p>0.05$; 12.7± 2.2 mmHg vs. 13.1 ± 2.3 mmHg, $p>0.05$; 13.6 ± 2.4 mmHg vs. 13.1 ± 2.3 mmHg, $p>0.05$, respectively)[8]. Whereas when CO_2 was added into the EVLP perfusate in blood-perfused lungs, 0% CO_2 decreased PAP, while 12% CO_2 increased PAP compared to 6% CO_2 control (15.5 ± 0.7 mmHg vs. 17.0 ± 0.7mmHg, $p<0.05$; 18.5 ± 0.8mmHg vs. 17.0 ± 0.7mmHg, $p<0.05$, respectively)[8]. Whereas when CO_2 was added into the EVLP perfusate in acellular buffer-perfused lungs, there was no significant difference in PAP with 0% and 12% CO_2 compared to 6% CO_2 control (13.1 ± 1.0 mmHg vs. 13.2 ± 1.1 mmHg, $p>0.05$; 13.1±1.1 mmHg vs. 13.2 ± 1.1 mmHg, $p>0.05$, respectively)[8].

3.2.2 Inert and Cytoprotective Gases (Ar, Xe, H_2)

Inert and cytoprotective gases such as argon (Ar), xenon (Xe), and hydrogen (H_2) were explored in five studies. Ar, Xe, and nitrogen (N_2) positive control were evaluated with 30% O_2 during 6 hours of porcine EVLP in Martens et al. 2016 [9]. PVR, P/F ratio, and PAW were not significantly different when Ar or Xe were administered (p>0.05 for all) compared to the controls [9]. In a follow-up study, Martens et al. 2017[10], compared the administration of Ar versus N_2 with 21% O_2 in a 4 hour porcine EVLP model. Ar administration had no effect on PVR, P/F ratio, and PAW compared to the N_2 control (732 dynes×sec×cm^{-5} vs. 628 dynes×sec×cm^{-5}, p=0.24; 370 vs. 365 (337-567), p=0.79; 18.5 cmH_2O vs. 19.5 cmH_2O, p=0.82, respectively[10]. Hees et al.[11] observed rats provided 5% CO_2 and 30% O_2 with either Ar or N_2 during 70 minutes of EVLP. Interestingly after 70 minutes of EVLP, Ar has significantly decreased increase in PVR and PAP, however did not significantly affect PAW compared to control when both 10^{-5} and 10^{-4} mmol/L phenylephrine were infused (10^{-5} mmol/L PVR: 124 ± 10% vs. 136 ± 7%, p=0.03; 10^{-4} mmol/L PVR: 136 ± 17% vs. 156 ± 6%, p=0.02; 10^{-5} mmol/L PAP: 121 ± 8% vs. 131 ± 10%, p=0.02; 10^{-4} mmol/L PAP: 132 ± 14% vs. 148 ± 15%, p=0.02; Airway pressure: 11.9 ± 3.1 mmHg vs. 13.4 ± 2.6 mmHg, p=0.38)[11].

Out of these studies, two looked at H_2 administration. Haam et al. 2015[12] used H_2 with 21% O_2 during 4 hours of EVLP, whereas Haam et al. 2018[13] used the same treatment and EVLP duration, but extended observation to 3 hours post-left lung transplantation in porcine models. Haam et al. 2015[12] found decreased PVR and PAP, but no significant difference in oxygen transfer capacity with H_2 treatment compared to control (p=0.011; p=0.019; p>0.05, respectively). Similarly, Haam et al. 2018[13] reported no significant difference in PVR during EVLP (p=0.432), but a significant decrease in PAP and a significant increase in dynamic compliance both during EVLP (p=0.027 and p=0.031, respectively) and after transplantation

EVLP ($p=0.020$ and $p=0.002$, respectively) with H_2 treatment compared to the control group[13]. Oxygen transfer capacity was not significantly different during EVLP, but it significantly improved after transplantation with H_2 treatment compared to the control group ($p>0.05$ and $p=0.033$, respectively)[13]. Lastly, the study by Noda et al. 2014[14] similarly utilized used H_2 with 21% O_2 during 4 hours of EVLP and additionally evaluated outcomes following left lung transplantation after 2 hours of EVLP and subsequent cold storage in a rodent model. During EVLP, H_2 administration was associated with decreased PVR, improved oxygenation, and increased compliance compared with the control group ($p < 0.05$ for all). Following transplantation, the H_2 -treated group also demonstrated significantly improved oxygenation compared with controls ($p < 0.05$)[14].

3.2.3 Inhaled Pharmacologic Therapies

Inhaled pharmacologic interventions were evaluated in five studies. Of these, two studies assessed beta 2 (β_2)-adrenoreceptor agonist intervention. In Kondo et al.[15], β_2-adrenoreceptor agonist was provided for 30 minutes (between minutes 20 and 50 of EVLP) in a 4 hour dog EVLP model. β_2-adrenoreceptor agonist compared to the control group was found to lower PVR, lower PAP, have no effect on partial pressure of oxygen, increase compliance, and lower PAW ($p=0.0068$; $p<0.001$; $p=0.12$; $p=0.026$; $p<0.001$, respectively)[15]. Hijiya et al.[16] similarly used a dog model; however, administered β_2-adrenoreceptor agonist four times (30 minutes in duration each) during two hours of EVLP, followed by observation 4 hours after left lung transplantation[16]. After 2 hours of EVLP, PVR, partial pressure of O_2, and compliance were not significantly different compared to the control group (1,425.8 ± 319.4 dynes x s x cm^{-5} vs. 1,422.1 ± 415.5 dynes x s x cm^{-5}, $p=0.473$; 492.8 ± 63.6 mmHg vs. 560.0 ± 61.6 mmHg, $p=0.064$; 23.1 ± 3.2 mL/cm H_2O vs. 24.8 ± 5.9 mL/cm H_2O, $p=0.291$, respectively)[16]. Whereas 4 hours post transplantation, PVR decreased, partial pressure of O_2 increased, and dynamic compliance increased compared to the control group (553.4 ± 219.9 dynes x s x cm^{-5} vs. 884.6 ±

146.6 dynes x s x cm^{-5}, $p=0.049$; 602.4 ± 71.6 mmHg vs. 95.6 ± 68.4 mmHg, $p<0.001$; 25.9 ± 3.1 mL/cm H_2O vs. 12.1 ± 1.4 mL/cm H_2O, $p<0.001$, respectively)[16].

N-acetylcysteine (NAC) was administered during 2 hours of porcine EVLP, followed by 6 hours of observation during and after porcine left lung transplantation in Yamada et al.[17]. Compared to controls, NAC did not affect PVR, oxygenation, and compliance ($p>0.05$; $p=0.053$; $p>0.05$, respectively)[17]. In Ohata et al.[18], fasudil was administered during the first five minutes of warm ischemia, then rat lungs underwent 3 hours of EVLP. PVR was decreased, oxygen partial pressure was increased, and compliance was increased compared to controls ($p<0.01$; $p<0.001$; $p<0.001$)[18]. In George et al.[19], administration of 2-S-amino-6-boronohexanoic acid (ABH) given in the first 10 minutes of EVLP was compared to administration after 3 hours of EVLP in a 4-hour human EVLP model. Between these two treatment regimen there was no difference in PAP ($p=0.5$) and partial pressure of O_2 ($p>0.05$), however administration of ABH in the first 10 minutes of EVLP led to a significant increase in compliance from baseline (73 ±17 ml/cm H_2O vs. 34 ±11 ml/cm H_2O, $p=0.001$) compared to ABH administration after 4 hours of EVLP (39 ±19 ml/cm H_2O vs. 46 ± 23 ml/cm H_2O, $p=0.8$)[19].

3.2.4 Anesthetics and Liquid Ventilation

Volatile anesthetics such as sevoflurane and liquid ventilation strategies using perfluorocarbon-based agents were explored in five studies. In Wang et al. 2018[20] the addition of 2% sevoflurane during the first 30 minutes of ventilation (gas mixture consisted of 6% O_2, 10% CO_2, and 84% N_2) was explored for a total rat EVLP duration of 3 hours. 2% sevoflurane administration was associated with no significant difference in PVR ($p=0.15$), no significant difference in oxygenation ($p=0.77$), consistent increased in compliance and decrease in PAW from 90-180 minutes of EVLP compared to the control group (90 minutes: 0.70 ± 0.21 mL x

cmH_2O^{-1} vs. 0.41 ± 0.08 mL x cmH_2O^{-1}, p=0.015; 6.53 ± 1.04 cmH_2O vs. 7.83 ± 0.75 cmH_2O, p=0.014; 120 minutes: 0.70 ± 0.19 mL x cmH_2O^{-1} vs. 0.44 ± 0.09 mL x cmH_2O^{-1}, p=0.001; 6.50 ± 1.11 cmH_2O vs. 7.48 ± 0.82 cmH_2O, p=0.001; 150 minutes: 0.69 ± 0.2 mL x cmH_2O^{-1} vs. 0.45 ± 0.09 mL x cmH_2O^{-1}, p<0.001; 6.48 ± 1.3 cmH_2O vs. 7.27 ± 0.48 cmH_2O, p<0.001; 180 minutes: 0.69 ± 0.22 mL x cmH_2O^{-1} vs. 0.45 ± 0.09 mL x cmH_2O^{-1}, p<0.001; 6.75 ± 1.43 cmH_2O vs. 7.18 ± 0.71 cmH_2O, p<0.001)[20]. 2% sevoflurane was further provided at the start of 4 hours of EVLP with a gas mixture containing 40% O_2 in Steinkuhler et al.[21] in a sheep model. In this model, P/F ratio was not significantly different whereas compliance increased compared to the control (437 vs. 347, p=0.056 and p=0.003, respectively)[21]. Junior et al.[22] provided 2% sevoflurane with 50% O_2 and intrapleural temperature (10°C-15°C) for 30 minutes prior to 8 hours of porcine lung EVLP. This treatment decreased PVR, increased P/F ratio, increased compliance, and decreased PAW compared to 50% O_2 and intrapleural temperature (10°C-15°C) alone (p<0.05; p<0.0001; p<0.0001; p<0.0001, respectively) [22].

Liquid ventilation was utilized during 6 hours of rat lung EVLP in Becerra et al.[23]. Liquid ventilation compared to gas ventilation has no effect on PVR, P/F ratio, and compliance (p=0.11; p>0.05; p=0.17, respectively)[23]. Loer et al.[24] similarly compared liquid ventilation to gas ventilation during rabbit lung EVLP. PVR, PAP, and partial pressure of O_2 were not statistically different between liquid ventilation and control (116 ± 20 H_2O/L/min vs. 101 ± 18 H_2O/L/min, no p value reported; 21.3 ± 4.3 cmH_2O vs. 19.2 ± 3.8 cmH_2O, no p value reported; 141 ± 7 torr vs. 145 ± 5 torr, no p value reported, respectively)[24].

3.3 Inflammatory, Oxidative Stress, and Apoptosis Markers

Inflammatory markers included tumor necrosis factor (TNF)α, pro-inflammatory interleukin (IL)-6, IL-1β, IL-8, and anti-inflammatory IL-10, with elevated levels indicating

increased inflammation. Oxidative stress was measured by malondialdehyde (MDA), myeloperoxidase (MPO) and/or protein carbonyl levels. Terminal deoxynucleotidyl transferase dUTP nick-end labeling positive (TUNEL+) stained cells, with increased amounts indicating increased apoptosis.

3.3.1 Vasoactive Gases (NO, CO, CO_2, O_2)

Out of the vasoactive gases, NO decreased TNFα in one study[6], had no effect on IL-1β in two studies[3,6], no effect on IL-6 in two studies[3,6], and no effect on IL-8 in one study[3]. Michaelsen et al.[3] compared 86% N_2, 8% CO_2, 6% O_2, with or without 200 ppm NO, reporting in tissue (at 1, 6, and 12 hours of EVLP) and in perfusate (before and after EVLP) no effect on IL-1β (In perfusate: 1.3 pg/mL vs. 0.1 pg/mL, p=0.285; 1.1 pg/mL vs. 0.3 pg/mL, p=0.485; 7.1 pg/mL vs. 2.5 pg/mL, p=0.685; In tissue: 61.3 pg/mL vs.100.4 pg/mL, p=0.628;96.1 pg/mL vs. 98.4 pg/mL, p=0.685, respectively), IL-6 (In perfusate: 5.9 pg/mL vs. 8.6 pg/mL, p=0.485; 226.8 pg/mL vs. 192.8 pg/mL, p>0.999; 1114.6 pg/mL vs. 626.9 pg/mL, p=0.342; In tissue: 33.9 pg/mL vs. 31.9 pg/mL, p=0.857; 155.6 pg/mL vs. 71.4 pg/mL, p=0.342, respectively), and IL-8 (In perfusate: 44.7 pg/mL vs. 44.7 pg/mL, p>0.999; 68.8 pg/mL vs. 54.7 pg/mL, p=0.200; 110.5 pg/mL vs. 73.8 pg/mL, p=0.485; In tissue: 38.3 pg/mL vs. 32.4 (pg/mL, p=0.114; 167.4 pg/mL vs. 111.5 pg/mL, p=0.685, respectively)[3]. In Dong et al. 2009[6], when 40 ppm NO with 60% O_2 was compared with 60% O_2 alone, TNFα decreased after EVLP (p=0.01) and after transplantation (p=0.01), had no effect on IL-1β (p>0.05), and no effect on IL-6 (p>0.05)[6]. CO on the other hand had no effect on TNFα in two studies[1,7], decreased IL-1β when CO was combined with 60% O_2 in one study[7], had no effect when CO was provided with 6% O_2 in another study [1], decreased IL-6 in two studies[1,7], had no effect on IL-8 in one study[1], increased IL-10 in tissue but not perfusate in one study[1], reduced MPO after transplant but not during EVLP in one study[1], and has no

effect on TUNEL+ cells[1]. Kalaf-Mussi et al.[1] compared 8% CO_2, 6% O_2, and balanced nitrogen with or without 500 ppm CO, in perfusate and in tissue at 6 hours of EVLP and 4 hours post transplantation, there was no significant effect on TNFα (In perfusate: 192.0 pg/mL vs. 263.50 pg/mL, p=0.52; 50.5 pg/mL vs. 43.8 pg/mL, p=0.52; In tissue: 34.9 pg/mg protein vs. 14.1 pg/mg protein, p=0.058; 10.9 pg/mg protein vs. 8.80 pg/mg protein, p=0.91, respectively), IL-1β (In perfusate only 6 hours of EVLP reported: 26.52 pg/mL vs. 25.57 pg/mL, p=0.56; In tissue: 134.9 pg/mg protein vs. 68.4 pg/mg protein, p=0.25; 585.1 pg/mg protein vs. 443.9 pg/mg protein, p=0.34, respectively), and IL-8 (In perfusate only 6 hours of EVLP reported: 795.2 pg/mL vs. 1269 pg/mL, p=0.17; In tissue only 4 hours of post transplant reported: 2420 pg/mg protein vs. 3123 pg/mg protein, p=0.46, respectively)[1]. Whereas IL-6 decreased with CO use in perfusate 4 hours post-transplant but not after EVLP in perfusate (4 hours post-transplant: 63.9 pg/mL vs. 128.9 pg/mL, p=0.03; 6 hours of EVLP: 567.2 pg/mL vs. 964.9pg/mL, p=0.91)[1]. IL-10 increased after in tissue 4 hours after transplantation but had no significant effect after 6 hours of EVLP (4 hours post-transplant: 32.0 pg/mL vs. 23.8 pg/mL, p=0.04; 6 hours of EVLP: 23.0 pg/mL vs. 19.4 pg/mL, p=0.25)[1]. Similarly, a decrease in MPO was seen in tissue 4 hours after transplantation but not after 6 hours of EVLP (4 hours post-transplant: 207.4 ng/mg protein vs. 532.5 ng/mg protein, p=0.009; 6 hours of EVLP: 147.8 ng/mg protein vs. 115.1 ng/mg protein, p=0.46) [1]. In Dong et al. 2013 [7], CO with 60% O_2 was compared with 60% O_2 alone, finding that CO with 60% O_2 decreased IL-1β (p=0.026) and IL-6 (p=0.0192), but had no significant effect on TNFα (p=0.087)[7].

Effect of O_2 varied by concentration, in one study 40% and 60% O_2 consistently decreased TNFα (p<0.01 for both), IL-1β (p<0.01 for both), and IL-6 (p<0.01 for both) after EVLP, but IL-1β (p<0.01) and IL-6 (p<0.01) remained lower after transplant only with 40% O_2,

while TNFα ($p<0.01$ for both) remained lower after transplant and reduced TUNEL+ cells ($p<0.01$ for both) in both 40% and 60% O_2 compared to 6% and 100% O_2[2]. Ellman et al.[4] additionally did not find any difference in MPO between 21-24% O_2 compared to 100% O_2 ($p=0.11$)[4]. Decreased inflammation and oxidative stress from CO_2 also differed by concentration, with one study showing decreased TNFα ($p<0.05$), IL-10 ($p<0.05$), and malondialdehyde (4.98 ± 0.44 nmol/mg protein vs. 5.81 ± 0.56 nmol/mg protein, $p<0.05$) in 10% CO_2 compared to 5% CO_2[25].

3.3.2 Inert and Cytoprotective Gases (Ar, Xe, H_2)

Ventilation with argon did not change TNF-α or IL-6 levels compared to controls in one study (Bronchoalveolar lavage: $p=0.11$ and $p=0.24$; Perfusate: $p=0.24$ and $p=0.09$, respectively)[10]. Hydrogen ventilation produced greater anti-inflammatory effects. Hydrogen therapy reduced TNF-α (0.102 ± 0.059 ng/ml vs. 0.361 ± 0.132, $p=0.0159$), IL-1β (193.6 ± 85.5 ng/ml vs. 309.7 ± 87.2, $p=0.0317$), IL-6 (1.524 ± 0.753 ng/ml vs. 4.112 ± 1.863, $p=0.0159$), and IL-8 (2.538 ± 1.833 ng/ml vs. 40.125 ± 19.382, $p=0.0195$) production but had no effect on MPO (54.5 ± 1.9 mU/mg vs. 51.5 ± 2.3 mU/mg, $p=0.1905$) after EVLP compared to the control in one study[12]. However, the effect of H_2 therapy after transplantation following EVLP was found to reduce IL-6 ($p=0.024$) and increased IL-10 ($p=0.037$), but had no effect on TNF-α ($p>0.05$) in another study[13]. With the shorter EVLP duration prior to transplantation used by Noda et al. 2014[14], H_2 administration significantly reduced TNFα, IL-1β, and IL-6 levels during EVLP and after transplantation compared with the control group ($p<0.05$ for all). The TUNEL staining results showed that H_2 treatment decreased apoptotic cells in lung tissue to $2.1 \pm 0.4\%$ compared to $3.4 \pm 1.6\%$ in control samples but the study did not provide a p-value[13].

3.3.3 Inhaled Pharmacologic Therapies

Administration of a β_2-adrenoreceptor agonist did not significantly alter IL-8 levels compared with controls ($p > 0.05$) in one study [15]. NAC treatment showed no difference in IL-1β ($p = 0.18$) and IL-8 (429 ± 162 pg/mL vs. 874 ± 544 pg/mL, $p=0.09$) compared to control lungs in one study[17]. For oxidative markers, although NAC treatment demonstrated no change in MDA ($p=0.17$) and protein carbonylation ($p=0.13$), there was a decrease in MPO activity (25.4 ± 6.4 U/mL vs. 34.2 ± 5.4 U/mL, $p=0.037$) compared to the control in one study[17]. One study showed that early compared to late treatment of nebulized ABH treatment did not produce any significant change in reactive oxygen species production ($p=0.5$)[19].

3.3.4 Anesthetics and Liquid Ventilation

Sevoflurane with 6% O_2 compared to the control was found to reduce TNF-α (0.59 ± 0.38 ng/mg vs. 1.50 ± 0.59 ng/mg, $p=0.02$), IL-8 (0.66 ± 0.28 ng/mg vs. 1.17 ± 0.32 ng/mg, $p=0.03$), and protein carbonylation (0.55 ± 0.11 nmol/mg vs. 1.17 ± 0.44 nmol/mg, $p=0.006$) but had no effect on IL-6 (1.58 ± 0.29 ng/ mg vs. 2.63 ± 1.06 ng/ mg, $p=0.11$) in one study[20]. In Junior et al.[22], intrapleural topical cooling and 50% O_2 with or without sevoflurane had no effect on TNF-α in perfusate and tissue ($p=0.063$ and $p>0.05$, respectively), IL-1β in perfusate and tissue ($p=0.0973$ and $p>0.05$, respectively), IL-10 in perfusate and tissue ($p=0.063$ and $p>0.05$, respectively), IL-1β in perfusate and tissue ($p=0.060$ and $p>0.05$, respectively), and TUNEL+ cells ($p>0.05$)[22]. In Steinkuhler et al.[21] compared the addition of sevoflurane with 40% O_2 compared to 40% O_2 alone, reporting no significant effect on TNF-α ($p>0.05$), IL-6 (difference in pre versus post EVLP gene expression: 0.708 vs. 3.385, $p=0.691$), and MPO (difference in pre versus post EVLP gene expression: -0.0198 vs. 0.0004, $p=0.222$)[21]. However, treatment compared to the control in this study significantly decreased IL-6 and IL-8 in perfusate but not in tissue (In perfusate: $p<0.05$ for both; In tissue: $p>0.05$ for both)[22]. Liquid ventilation compared to

gas ventilation produced no significant differences in TNF-α ($p>0.05$), IL-6 ($p>0.05$), or TUNEL staining ($p>0.05$) in one study[23].

3.4 Edema, metabolic homeostasis, and histological lung injury score

Lung injury was assessed by reporting of increased edema formation, lactate, glucose consumption, and histological lung injury score. Edema formation consisted of either wet-to-dry lung weight ratio (W/D) or weight gain.

3.4.1 Vasoactive Gases (NO, CO, CO_2, O_2)

CO ventilation compared to control during 6 hours of EVLP followed by observation 4 hours post-transplant, reduced W/D ratio but had no significant effect on histological lung injury score both during EVLP and after transplantation (EVLP: $p=0.027$ and $p=0.206$; Post-transplant: $p=0.003$ and $p=0.643$, respectively)[1]. Ventilation with 60% O_2 with CO compared to 60% O_2 alone during 1 hour of EVLP followed by 1 hour post-transplant decreased W/D ratio during EVLP ($p=0.0356$), though the effect was not maintained after transplantation ($p>0.05$)[7]. Similarly, combining 60% O_2 and 40ppm NO reduced the W/D ratio after EVLP ($p<0.003$) but showed no difference following transplantation ($p>0.05$)[6]. 200ppm NO exposure with a gas mixture containing 6% O_2 compared to the control did not significantly affect W/D ratio (5.5 ± 0.4 vs. 5.1 ± 0.9, $p=0.628$), lactate levels (6.0 ± 2.1 vs. 6.8 ± 2.5, $p=0.694$), and histological lung injury score ($p>0.05$)[3]. A follow-up clinical pilot demonstrated that 6% O_2 with compared to without 20 or 80 ppm NO modestly lowered lung weight pre to post 6 hour EVLP (20 ppm NO: 3881.3g to decreased to unspecified weight vs. 80 ppm NO: 1406.9 g to 1604.3 g vs. Control: 1615.6 g to 2255.6 g, p value no reported,) but had minimal effect on histological injury score (4.0 to 2.3 vs. 4.3 to 2.3 vs. 4.1 to 1.5, p value no reported, respectively), but significance was not measured[5].

While hypercapnic ventilation (10% CO_2) compared to 5% CO_2 also decreased W/D ratio (5.97 ± 1.01 vs. 7.87 ± 0.24, p<0.05) and improved histological injury scores (3 vs. 5, p<0.05) in one study[25]. In one study, exposure to 21–24% O_2 produced lower W/D ratios compared to 100% O_2 (p=0.002)[4]. In another study, lungs perfused with 40% and 60% O_2 showed less edema on histological analysis compared to 6% and 100% O_2 groups, however significance was not measured[2].

3.4.2 Inert and Cytoprotective Gases (Ar, Xe, H_2)

Ventilation with Ar did not alter the W/D ratio in two studies[9,10] and Ar and Xe did not alter histological score in terms of alveolar macrophages, neutrophils, alveolar necrosis and bronchial inflammation in one study[9]. Martens et al 2017[10] observed no significant difference in W/D ratio (7.5 vs. 7.4, p=0.47) in Ar ventilated lung compared to the control[10]. Hydrogen significantly lowered the W/D ratio after EVLP (5.077 ± 0.268 vs. 5.715 ± 0.557, p=0.0400) in one study[12], did not have significant effects on W/D ratio after transplant (1.766 ± 0.078 vs. 2.833 ± 0.354, p>0.05) in another study[13], and lowered histologic lung injury score in Haam et al. 2015[12] and in Haam et al 2018[13] (0.2 ± 0.2 vs. 0.9 ± 0.1, p=0.0358; 0 or 1 vs. 2 or 3, p=0.0159, respectively). Additionally, in Noda et al. 2014, H_2 treatment significantly reduced lactate compared to the control (p<0.01) during EVLP[14].

3.4.3 Inhaled Pharmacologic Therapies

Treatment with a β_2-adrenoreceptor agonist decreased W/D ratio and improved histologic architecture, in terms of macroscopic lung appearance and presence of edematous tissue in two studies[15,16]. In Kondo et al.[15], β_2-adrenoreceptor agonist decreased W/D ratio compared to the control group (5.72 ± 0.88 vs. 8.78 ± 0.61, p=0.0027). In Hijiya et al.[16], a β_2-adrenoreceptor agonist decreased W/D 4 hours post-transplantation (6.4 ± 0.5 vs. 10.2 ± 1.6, p<0.001). Nebulized fasudil compared the control also decreased W/D ratio (p<0.001) and yielded

improved histologic preservation described by attenuated edema and hemorrhage in one study[18]. By contrast, NAC treatment during porcine EVLP led to stable histologic scores without significant differences from control lungs (4.50 ± 0.84 vs. 5.0 ± 0.70, p=0.32)[17].

3.4.4 Anesthetics and Liquid Ventilation

Lungs ventilated with sevoflurane demonstrated reduced edema in two studies[20,22] and reduced histologic lung injury score in one study[20]. In Wang et al. 2018[20], edema was indicated by weight gain and histological score was based on perivascular edema which was reduced with sevoflurane compared to the control (0.52 ± 0.06 g vs. 0.72 ± 0.09 g, p=0.044; 0.47 ± 0.07 vs. 0.58 ± 0.09, p=0.036, respectively). In Junior et al [22]. edema was similarly measured by lung weight and was found to be decreased when sevoflurane was provided 30 minutes prior to EVLP with intrapleural topical cooling compared to intrapleural topical cooling alone (420 ± 58 g vs. 760 ± 309 g, p<0.001)[22]. Liquid ventilation no significant changes in W/D ratio (p=0.16) and lactate (p>0.05) in one study[23].

References

1. Kalaf-Mussi R, Nakajima D, Chen M, et al. Therapeutic Use of Carbon Monoxide in Ex-Vivo Lung Perfusion in Donor With Prolonged Cold Ischemia. *J Surg Res*. 2024;301:315-323.
2. Noda K, Tane S, Haam SJ, et al. Optimal ex vivo lung perfusion techniques with oxygenated perfusate. *J Heart Lung Transplant*. 2017;36(4):466-474.
3. Michaelsen VS, Ribeiro RVP, Ali A, et al. Safety of continuous 12-hour delivery of antimicrobial doses of inhaled nitric oxide during ex vivo lung perfusion. *J Thorac Cardiovasc Surg*. 2022;163(3):841-849.e1.
4. Ellman PI, Alvis JS, Tache-Leon C, et al. Hyperoxic ventilation exacerbates lung reperfusion injury. *J Thorac Cardiovasc Surg*. 2005;130(5):1440.e1-1440.e8.
5. Hartwig MG, Klapper JA, Poola N, et al. A Randomized, Multicenter, Blinded Pilot Study Assessing the Effects of Gaseous Nitric Oxide in an Ex Vivo System of Human Lungs. *Pulm Ther*. 2023;9(1):151-163.
6. Dong BM, Abano JB, Egan TM. Nitric oxide ventilation of rat lungs from non-heart-beating donors improves posttransplant function. *Am J Transplant*. 2009;9(12):2707-2715.
7. Dong B, Stewart PW, Egan TM. Postmortem and ex vivo carbon monoxide ventilation reduces injury in rat lungs transplanted from non-heart-beating donors. *J Thorac Cardiovasc Surg*. 2013;146(2):429-436.e1.
8. Yamamoto Y, Nakano H, Ide H, et al. *J Physiol* 2001:91.
9. Martens A, Montoli M, Faggi G, et al. Argon and xenon ventilation during prolonged ex vivo lung perfusion. *J Surg Res*. 2016;201(1):44-52.
10. Martens A, Ordies S, Vanaudenaerde B, et al. A porcine ex vivo lung perfusion model with maximal argon exposure to attenuate ischemia-reperfusion injury. *Med Gas Res*. 2017;7(1):28-36.
11. Hees JE, Cleveland WJ, Balzer C, Riess ML. Inhaled argon dilates pulmonary vasculature in rat isolated lungs. *Can J Physiol Pharmacol*. 2025;103(1):29-35.
12. Haam S, Lee S, Paik HC, et al. The effects of hydrogen gas inhalation during ex vivo lung perfusion on donor lungs obtained after cardiac death. *Eur J Cardio-thorac Surg*. 2015;48(4):542-547.
13. Haam S, Lee JG, Paik HC, Park MS, Lim BJ. Hydrogen gas inhalation during ex vivo lung perfusion of donor lungs recovered after cardiac death. *J Heart Lung Transplant*. 2018;37(10):1271-1278.
14. Noda K, Shigemura N, Tanaka Y, et al. Hydrogen preconditioning during ex vivo lung perfusion improves the quality of lung grafts in rats. *Transplant*. 2014;98(5):499-506.
15. Kondo T, Chen F, Ohsumi A, et al. β2-Adrenoreceptor Agonist Inhalation During Ex Vivo Lung Perfusion Attenuates Lung Injury. *Ann Thorac Surg*. 2015;100(2):480-486.
16. Hijiya K, Chen-Yoshikawa TF, Kondo T, et al. Bronchodilator Inhalation During Ex Vivo Lung Perfusion Improves Posttransplant Graft Function After Warm Ischemia. *Ann Thorac Surg*. 2017;103(2):447-453.

17. Yamada Y, Iskender I, Arni S, et al. Ex vivo treatment with inhaled N-acetylcysteine in porcine lung transplantation. *J Surg Res*. 2017;218:341-347.
18. Ohata K, Chen-Yoshikawa TF, Menju T, et al. Protective Effect of Inhaled Rho-Kinase Inhibitor on Lung Ischemia-Reperfusion Injury. *Ann Thorac Surg*. 2017;103(2):476-483.
19. George TJ, Arnaoutakis GJ, Beaty CA, et al. A physiologic and biochemical profile of clinically rejected lungs on a normothermic ex vivo lung perfusion platform. *J Surg Res*. 2013;183(1):75-83.
20. Wang X, Parapanov R, Francioli C, et al. Experimental ex vivo lung perfusion with sevoflurane: Effects on damaged donor lung grafts. *Interact Cardiovasc Thorac Surg*. 2018;26(6):977-984.
21. Steinkühler T, Yang S, Hu MA, et al. Ex Vivo Optimization of Donor Lungs with Inhaled Sevoflurane during Normothermic Ex Vivo Lung Perfusion (VITALISE): A Pilot and Feasibility Study in Sheep. *Int J Mol Sci*. 2024;25(4).
22. Junior ERB, Wang A, Ribeiro RVP, et al. The combination of postmortem sevoflurane ventilation and in situ topical cooling provides improved 6 hours lung preservation in an uncontrolled DCD porcine model. *J Heart Lung Transplant*. 2025;44(5):780-792.
23. Becerra D, Linge H, Jeffs S, Roberts S, Jane O, Ott HC. Liquid Ventilation Reconditions Isolated Rat Lungs Following Ischemia-Reperfusion Injury. *Tissue Eng Part A*. 2022;28(21-22):918-928.
24. Loer SA, Schlack W, Ebel D, Tarnow J. Laboratory Investigations Effects of Partial Liquid Ventilation on Regional Pulmonary Blood Flow Distribution of Isolated Rabbit Lungs.
25. Wu SY, Wu CP, Kang BH, Li MH, Chu SJ, Huang KL. Hypercapnic acidosis attenuates reperfusion injury in isolated and perfused rat lungs. *Crit Care Med*. 2012;40(2):553-559.
